# Differential Nutrient Limitation of Soil Microbial Biomass and Metabolic Quotients (*q*CO_2_): Is There a Biological Stoichiometry of Soil Microbes?

**DOI:** 10.1371/journal.pone.0057127

**Published:** 2013-03-19

**Authors:** Wyatt H. Hartman, Curtis J. Richardson

**Affiliations:** Duke University Wetland Center, Nicholas School of the Environment, Duke University, Durham, North Carolina, United States of America; Missouri University of Science and Technology, United States of America

## Abstract

**Background:**

Variation in microbial metabolism poses one of the greatest current uncertainties in models of global carbon cycling, and is particularly poorly understood in soils. Biological Stoichiometry theory describes biochemical mechanisms linking metabolic rates with variation in the elemental composition of cells and organisms, and has been widely observed in animals, plants, and plankton. However, this theory has not been widely tested in microbes, which are considered to have fixed ratios of major elements in soils.

**Methodology/Principal Findings:**

To determine whether Biological Stoichiometry underlies patterns of soil microbial metabolism, we compiled published data on microbial biomass carbon (C), nitrogen (N), and phosphorus (P) pools in soils spanning the global range of climate, vegetation, and land use types. We compared element ratios in microbial biomass pools to the metabolic quotient *q*CO_2_ (respiration per unit biomass), where soil C mineralization was simultaneously measured in controlled incubations. Although microbial C, N, and P stoichiometry appeared to follow somewhat constrained allometric relationships at the global scale, we found significant variation in the C∶N∶P ratios of soil microbes across land use and habitat types, and size-dependent scaling of microbial C∶N and C∶P (but not N∶P) ratios. Microbial stoichiometry and metabolic quotients were also weakly correlated as suggested by Biological Stoichiometry theory. Importantly, we found that while soil microbial biomass appeared constrained by soil N availability, microbial metabolic rates (*q*CO_2_) were most strongly associated with inorganic P availability.

**Conclusions/Significance:**

Our findings appear consistent with the model of cellular metabolism described by Biological Stoichiometry theory, where biomass is limited by N needed to build proteins, but rates of protein synthesis are limited by the high P demands of ribosomes. Incorporation of these physiological processes may improve models of carbon cycling and understanding of the effects of nutrient availability on soil C turnover across terrestrial and wetland habitats.

## Introduction

Variation in heterotrophic microbial metabolism poses a critical uncertainty in our current understanding of soil carbon (C) cycling in terrestrial and wetland soils, and improved understanding of microbial mediation of soil C and nutrient cycling is needed to predict ecosystem responses to human alteration of land use, climate, and nutrient availability [1], [2], [3], [4]. Soil carbon turnover in terrestrial and wetland ecosystems may closely depend on the availability of major nutrients like nitrogen (N) and phosphorus (P), and ratios of these elements relative to microbial demand strongly influences C and nutrient mineralization during decomposition [2], [5], [6]. However, the processes governing the relative demand of microbes for C, N, and P in soils are poorly understood, and little data is available to characterize variation in the stoichiometry and metabolism of soil microbes [6], [7], [8].

At global scales, the relative demand of soil microbes for C, N, and P are thought to occur in broadly fixed ratios, reflected by patterns in the elemental stoichiometry of both microbial biomass pools and soil enzyme activities [7], [9], [10]. However, in ecosystem studies similar large-scale stoichiometric patterns may obscure considerable variability among habitats [11] and stoichiometric variation is routinely observed in terrestrial and aquatic ecosystems among habitats, and among species of autotrophs and heterotrophs [8]. We might expect similar stoichiometric variation in soil microbes, given widely reported differences in the C∶N∶P biomass ratios of aquatic and cultured microbes among ecosystems, habitats, and taxonomic groups [12], [13], [14], [15]. Variation in the stoichiometry of microbes may be coupled with differences in growth rates like other heterotrophs [14], and might influence C use efficiency during decomposition in terrestrial ecosystems [6].

Metabolic relationships between carbon and nutrient cycling in heterotrophs can be understood as a function of the biochemical composition of the cellular machinery. Particularly, the Growth Rate Hypothesis (GRH) describes a relationship between cellular growth rate and P concentrations that results from the requirement of growing cells for P-rich ribosomes to produce new proteins [16], [17]. Relationships between organismal stoichiometry and growth rate described by the GRH appear consistently across heterotrophs and autotrophs spanning several orders of magnitude in size, and have broad implications at ecosystem scales (called Biological Stoichiometry) linked to trophic status and functional differentiation of communities and whole ecosystems [11], [16], [18], [19], [20], [21], [22], [23], [24]. While relationships between stoichiometry and metabolism are essentially untested in soils [8], [25], biochemical mechanisms described by the GRH may suggest microbial metabolism in soils could be particularly sensitive to ecosystem P availability.

The availability of phosphorus may be particularly important for the growth and metabolism of microbes in soils, with significant implications for decomposition and global carbon cycling. On average, the relative P demand of soil microbial biomass (C∶N∶P = 60∶7∶1) is considerably greater than the relative availability of P in soils (C∶N∶P = 186∶13∶1) and plant litter (C∶N∶P = 3144∶45∶1) inputs [10]. Moreover, the global stoichiometry of enzyme activities (C∶N∶P = 1∶1∶1) in terrestrial and aquatic ecosystems [9] may suggest microbial allocation to P uptake is considerably greater than that of C and N when compared to requirements for the growth of biomass. Although the influence of P availability on soil C cycling has been understudied compared to variation in N availability and C∶N ratios, recent findings indicate that P availability may influence soil C cycling even in ecosystems traditionally considered to be N limited [2], [6], [26], [27], [28].

To assess whether the influence of P availability on soil C cycling might reflect underlying metabolic mechanisms like the GRH, we collected all available data from published studies to evaluate the effects of N and P availability on the stoichiometry, biomass, and metabolic rates of soil microbes. Our cross-ecosystem dataset included measurements of soil and microbial C, N, and P pools, and C mineralization rates in soils spanning global variation soil conditions and nutrient availability in terrestrial and wetland ecosystems, and we explicitly considered study location factors of climate, land use, and vegetation as predictor variables. Soil C mineralization data were obtained only from controlled laboratory incubations in studies that simultaneously measured microbial element pools. We indexed the metabolism of microbes using the metabolic quotient *q*CO*_2_* [29], calculated as the rate of C mineralization per unit of microbial biomass C, and also referred to as mass specific respiration [30]. Where data were available, we also assessed the influence of inorganic P availability [31] and soil pH on microbial stoichiometry, growth, and metabolism.

## Results and Discussion

### Global variation in the stoichiometry of soil resources

**Figure 1:**
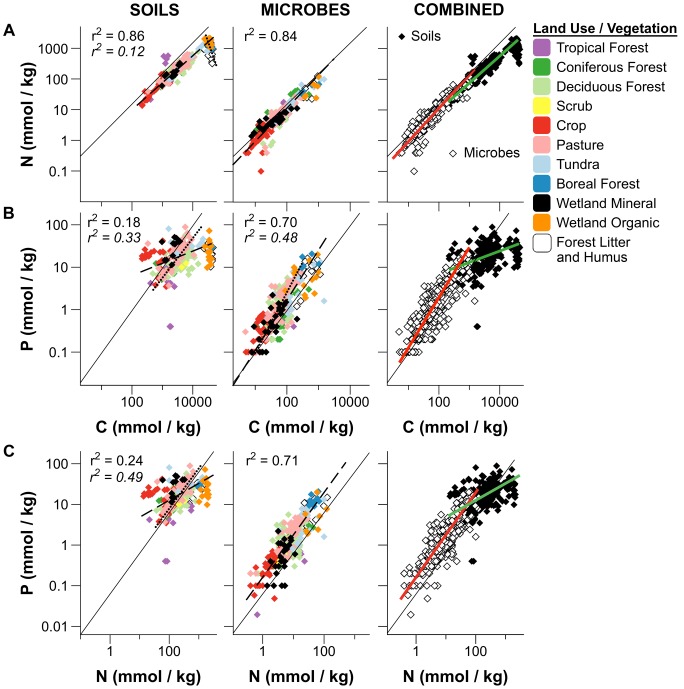
Global stoichiometric scaling of C, N, and P contents of soil and microbial biomass pools. Relationships in plots show variation in A) C∶N ratios B) C∶P ratios C) N∶P ratios of soils, microbial biomass, and combined data. Data were log_10_ transformed transformed to improve normality and plotted to express size dependent relationships in comparison to the Redfield (1958) ratios (solid black lines). Dashed lines are regression fits for all global soils, with correlation coeficients in plain text and parameters estimated by SMA given in Table 1. Global relationships were compared with fits obtained using different subsets of habitat types, and where slopes were significantly different we plotted fits as dotted lines, with correlation coeficients given in italics. Soil C∶N scaling (A) was different in litter and organic soils (wetland organic, boreal forest, and humic horizons), while forest and pasture soils were different from global relationships in soil and microbial C∶P (B) and soil N∶P (C). SMA regression parameters for these relationships using subsets of our data are given in Table S1.

Although a previous study of forest and grasslands suggested that pools of soil C, N, and P are closely linked with allometric relationships describing nearly fixed stoichiometry [10], our broader data set indicated considerable differences in the C∶N∶P stoichiometry of terrestrial and wetland soils, especially with respect to the relative availability of P. Carbon and nitrogen pools appeared closely coupled in soils (Fig. 1A), although we found some evidence that C and N pools did not increase isometrically as in a previous study [10]. Across all global soils, the allometric slope of the relationship between soil C and N was 0.88 (Table 1), which falls below the 1∶1 line representing constant C∶N ratios (Fig. 1A). This relationship represents a subtle decline in the relative availability of N with increasing accumulation of soil C pools. However, we note that soil C and N scaling was significantly different in litter layers and organic soils, where soil C∶N ratios increased dramatically with soil C (Fig. 1A, Fig. S1). Although scaling of C and N appeared to be isometric (slope = 1) when considering only forest and pasture soils (Table S1), closer analysis revealed a slight, but significant increase in C∶N ratios with increasing C in forest and pastures (Fig. S1, Table S1) like the global pattern.

**Table 1:** Summary of SMA regressions of log_10_-transformed C, N, and P contents in soil and microbial pools, along with predictors of soil C mineralization (CO_2_) and microbial metabolism (*q*CO_2_).

Analysis	*x*	*y*	*n*	r^2^	Int.	Slope	x∶y ratio	x∶y mean			CV
Soil allometry	*C	N	280	0.86	−0.77	**0.88**	C∶N	18.8	±	0.8	0.7
	*C	P	261	0.18	0.23	**0.29**	C∶P	445	±	43	1.6
	*N	P	261	0.24	0.27	**0.42**	N∶P	21.4	±	1.7	1.3
Microbial allometry	MBC	MBN	237	0.84	−1.11	**1.08**	mC∶N	10.7	±	0.6	0.8
	*MBC	MBP	298	0.70	−2.07	**1.18**	mC∶P	87.2	±	13.2	2.6
	MBN	MBP	267	0.71	−0.79	1.04	mN∶P	9	±	1.2	2.2
Microbial biomass	C	MBC	289	0.75	−1.34	**0.88**	MBC∶C	2.01%	±	0.08	0.6
	N	MBC	266	0.73	−0.52	0.99					
	*P	MBC	247	0.132	0.08	**1.37**					
Respiration and metabolism	MBC	CO_2_	92	0.7	−4.17	**1.57**					
	**mC∶P	*q*CO_2_	92	0.21	1.97	**−1.15**					
	**Pi	*q*CO_2_	38	0.44	0.51	**0.65**					

Bivariate relationships were significant (P<0.001) for all relationships shown. Slopes significantly different from one (P>0.05) are shown in boldface font. Slopes not different from one (not bold) indicate an isometric (linear) relationship among parameters. The geometric mean and standard errors (SE) of stoichiometric ratios (x∶y ratio, x∶y mean) are given for reference, but are not representative where allometric slopes are different than 1. The coefficient of variation (CV) of these stoichiometric ratios is provided as a dimensionless index of dispersion about the mean. Single asterisks (*) indicate where different slopes are observed by considering only forest and pasture soils (Table S1), and ** indicates relationships fit for all soils excluding litter and humus.

The stoichiometry of soil phosphorus appeared considerably more variable than N across global soils, with much wider divergence in soil C∶P and N∶P ratios from the Redfield ratios (Fig. 1, Fig. S1). In contrast to previous findings in forests and pastures [10], our analysis across a broader range of global ecosystems showed marked departure in soil C∶P and N∶P from the previously observed isometric relationships (Fig. 1B–C, Table 1, Table S1). The additional ecosystem types included in our analysis could be considered “disturbed” outliers, with lower C∶P in crops due to tillage increasing soil P, and higher C∶P in boreal forests, wetland organic soils, and litter resulting from relatively undecomposed substrates. However, these “outlier” ecosystems appear as endpoints in nearly continuous relationships in soil C∶P and N∶P ratios, which increased directly as a function of soil C (Fig. S1, Table S2). This dependence of soil C∶P and N∶P ratios on soil C may arise from dilution of soil P concentrations with soil C accumulation, as total soil P does not increase concomittantly with soil C to nearly the extent that N does (Fig. 1).

Variation in N∶P ratios across global soils is shaped by fundamental differences in the ecosystem sources of soil N (from atmospheric fixation by soil heterotrophs) and P (from mineral weathering) [32]. While soil N pools appeared closely linked with soil C accumulation, soil P pools were only weakly related to soil C, and were highly variable within and among ecosystem types (Fig. 1). Dilution of soil P concentrations by soil C (and N) accumulation appeared to be a primary driver of variation in soil N∶P ratios, which were tighly linked with C∶P ratios across all global soils (r^2^ = 0.88), but only weakly varied with C∶N ratios in leaf litter and soil humic horizons (Fig, S2, Table S2).

Although comparison of soil stoichiometry to the Redfield ratios might suggest that soil N appears more consistently limiting across global ecosystems than soil P (Fig. 1, Fig. S1), we note that the autotroph-based Redfield ratios may not appropriately describe microbial stoichiometry [14]. Soil microbial biomass N∶P ratios (9.0∶1) were considerably lower (more P rich) than the Redfield ratio (16∶1) or N∶P ratios of soils (21∶1, Table 1). Although this excess of P compared to the Redfield ratio could be interpeted as luxury uptake (reflecting N limitation), accumulation of excess P by soil microbes appears to be of minor importance, and coincides primarily with extreme P limitation [33]. We posit that microbial P demand is intrinsically greater than the Redfield ratio based on their small size and higher rates of metabolism compared to multicellular organisms [16], [18], [34], [35], [36]. If microbial biomass P quotas are intrinsically greater than the Redfield ratio, P availability in soils would appear to be limiting to microbes relative to N across a considerably broader range of ecosystem types than suggested by the Redfield ratio, including ecosystems typically considered to be N limited with respect to plant growth (Fig. S1C).

### Variation in soil microbial stoichiometry

In comparison to the wide variation we observed in the C∶N∶P ratios of soils, the stoichiometry of soil microbes appeared to be largely constrained (Fig. 1), although our results suggest potentially important biological and ecological sources of variation. Scaling relationships between C and N pools were generally similar in microbes and soils, although the slopes of these relationships were somewhat different (Table 1). In contrast, scaling of P pools with C and N differed dramatically among soils and microbes, with C∶P and N∶P ratios appearing more isometric (slopes closer to the 1∶1 line) in microbes than in soils (Fig. 1, Table 1). Similar to the results of a previous study [10], we found that microbial C∶N, C∶P, and N∶P ratios were not correlated with the corresponding element ratios in soils (Fig. S3, Table S2). The relative inflexibility of microbial biomass compared to highly variable resource ratios in soils has been suggested to reflect a homeostatic maintenance of microbial C∶N∶P ratios with nearly fixed stoichiometry [10]. While our results appear to similarly illustrate Resource Homeostasis in soil microbes, we emphasize that this need not imply soil microbial stoichiometry is inherently biologically or ecologically inflexible (i.e. Strict Homeostasis [37]).

Our results directly showed that the C∶N∶P stoichiometry of soil microbes does not occur in strictly fixed isometric ratios. At global scales, we observed slight increases in the proportions of N and P with increasing soil microbial biomass C pools, as indicated by allometric slopes significantly greater than one (Fig. 1, Table 1). This size-dependent effect was particularly pronounced for microbial P contents, and even more prominent in forest and pasture soils (Fig. 1, Table S1). These increases in average cellular N and P content with increasing microbial biomass in soils may indicate that microbial nutrient use efficiency (NUE) declines as microbial population size increases, a finding broadly analogous to size-dependent decreases in NUE (especially with respect to P) in aquatic bacteria [38]. However, we did not find a significant size-dependent scaling relationship between microbial N and P pools (allometric slope = 1.04) analogous to those observed in aquatic microbes [38] and across species of plants and animals [11], [16], [23], [39], [40].

**Figure 2:**
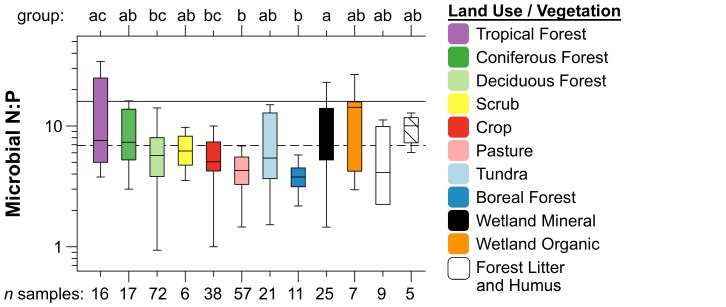
Differences in N∶P stoichiometry of soil microbial biomass among global vegetation and land use categories. Letters on x-axis above the plot show group differences among vegetation types (using Tukey's tests), and number of samples for each vegetation type are given on the lower x-axis. Overall variance described by vegetation (R^2^ = 0.193, p<0.001) was determined using a general linear model. Solid horizontal line is the Redfield (1958) ratio N∶P = 16∶1, dashed line is average microbial N∶P (6.9) reported in [10].

Stoichiometric analysis of soil microbial pools differs fundamentally from that of higher organisms, as the unit of observation is the elemental average of mixed communities rather than individual organisms [41], [42]. In stoichiometric analysis of ecosystem element pools, global scaling relationships reflecting broad stoichiometric constraints may obscure important variation among habitats [11], which may reflect both shifts in species composition, as well as the coexistence of species mixtures with different stoichiometry [41], [42]. We found that although microbial stoichiometry appeared on average to converge on broadly constrained ratios at a global scale, the N∶P ratios of soil microbes varied significantly among vegetation and land use types across global soils and litter layers (Fig. 2). Differences in soil microbial stoichiometry have also been observed previously with land use change in temperate forest and grassland ecosystems [10], [43], [44], [45], [46], [47]. While we note that the stoichiometry of microbes is highly variable within habitat types, our results show vegetation and land use may broadly influence the N∶P ratios of soil microbes across the global range of terrestrial ecosystems (Fig. 2).

Our analyses did not clearly identify factors associated with vegetation and land use types that might underlie variability in microbial stoichiometry across global soils. While vegetation and land use categories were associated with soil C pools and soil stoichiometry (Table S3), these factors were not correlated with microbial stoichiometry, nor were any other soil chemical or biological parameters (Table S4). We suggest instead that the differences we observed in microbial N∶P ratios among land use and vegetation types might be linked with variation in size-dependent scaling relationships.

**Figure 3:**
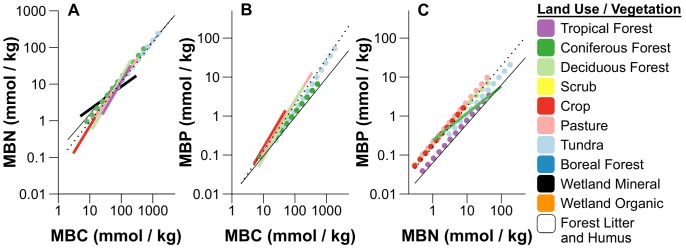
Estimated differences in stoichiometric scaling of microbial C, N, and P by land use/vegetation categories. Estimated SMA regression fit lines for each land use and vegetation category are shown to express habitat level differences in scaling of microbial biomass A) C∶N ratios, B) C∶P ratios, and C) N∶P ratios, with data log_10_ transformed for normality. Bold lines are colored by land use and vegetation category, and treatments without significant fits are not shown. Colored solid lines indicate relationships where slopes were not equal to 1, while slopes not significantly different from 1 are are displayed as bold colored dotted lines. Thin black dotted lines show the regression fits for all groups combined (the same as in Fig. 1), while thin black solid lines indicate the Redfield ratios (C∶N∶P = 106∶16∶1). Individual plots for each regression fit by land use and vegetation categories are given in Figure S4, with parameters estimated by SMA provided in Tables S3, S4, S5, along with results of intercept and slope tests, and multiple comparisons of these parameters.

The stoichiometric scaling relationships of microbes differed significantly among land use and vegetation types (Tables S5, S6, S7, Fig. S4), and we directly compared habitat specific differences in these scaling relationships (Fig. 3). Size-dependent scaling of soil microbial C∶N and C∶P (but not N∶P) like that observed in global relationships (Fig. 1, Table 1) was present in some but not all habitats, with variation in the degree of deviation of allometric slopes from the isometric Redfield ratios among land use and vegetation types (Fig. 3). When comparing the slopes of stoichiometric relationships among land use and vegetation categories, size-dependent scaling relationships of soil microbial biomass C∶N and C∶P appeared to be somewhat asymptotic to the Redfield ratio as the size of the biomass pools increased (Fig. 3A–B).

Size-dependent slopes of C∶N and C∶P scaling relationships appeared closely coupled in forests, crops, and pasture soils (Fig. 3A–B, Tables S5, S6, S7), and these parallel decreases in NUE of N and P appeared to “cancel out” size-dependent relationships in microbial N∶P scaling (Fig. 3, Fig. 1). However, we note that while the slopes of microbial C∶N scaling relationships among habitats approached the Redfield ratio from below (higher C∶N) as biomass C increased, microbial C∶P scaling approached the Redfield ratio from above (lower C∶P). These generally higher C∶N and lower C∶P ratios (relative to Redfield) in habitats where C∶N and C∶P scaling was size-dependent could help explain variation in microbial N∶P ratios by habitat, despite their lack of size-dependent relationships (Fig. 3).

Global scale variation in the stoichiometry of autotrophs may reflect broad patterns based on climate [40], [48], [49], [50], and we found some indication of a similar climatic influence on the stoichiometry of microbes. Although microbial stoichiometry did not vary closely with latitude or climate categories (Table S4), we found a significant effect of climate on stoichiometric scaling relationships of microbes (Tables S8, S9, S10) like that of vegetation and land use (Fig. 3). While our climate categories did not represent physiochemical factors like soil temperature or moisture, these factors may appear linked with seasonal variation in microbial stoichiometry within habitats [51], [52], [53], [54], [55]. Variation in microbial stoichiometry could also be linked with other factors not captured by our dataset, such as soil C quality [7], [56] or microbial communities [57], [58], [59].

Our results showed important variation in the stoichiometry of soil microbes, including size-dependent scaling of C∶N and C∶P, and differences in the N∶P ratios of soil microbial biomass among land use and vegetation types that might arise from size-dependent differences in NUE among habitats. We also found some indication of non-homeostatic responses to soil stoichiometry in a few vegetation and climate categories, albeit with small sample sizes (Tables S11, S12). These results generally indicate that the stoichiometry soil microbes is not inherently fixed, but instead may exhibit some stoichiometric flexibility like cultured and aquatic microbes, which similarly show non-homeostasis among habitats and size-dependent scaling patterns [12], [14], [15], [38], [42], [60].

### Limitation of microbial biomass pool size

**Figure 4:**
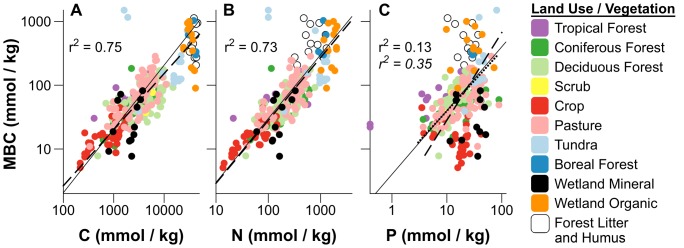
Scaling of soil microbial biomass C (MBC) with soil C, N, and P pools. Relationships in plots show variation in MBC with A) soil C, B) soil N, and C) soil P. Outliers from the general relationship between MBC and soil C in A), including floodplain mineral soils [95] and arctic tundra [96] were removed prior to fitting regressions, and data were log_10_ transformed to improve normality. Solid lines are the 1∶1 isometric lines based on the geometric mean ratio of each scaling relationship. Dashed lines are regression fits for all global soils, with correlation coeficients in plain text and parameters estimated by SMA given in Table 1. Global relationships were compared with fits obtained using only data from forests and pastures, and where slopes were significantly different from all combined treatments we plotted fits as dotted lines, with correlation coeficients given in italics. SMA regression parameters fit using only forest and pasture soils are given in Table S1. Regressions were also tested seperately with only litter and organic soils data, but were these relationships were not significant.

Although we found the relative availability of P varied more dramatically than N across global soils, pools of microbial biomass carbon (MBC) were not closely related to soil P, but rather more closely reflected C and N pools in soils (Fig. 4). Although MBC was closely related to both soil C and soil N, the relationship between MBC and N was linear, while increases in MBC with soil C were non-linear, slightly lagging accumulation of soil C (Fig. 4A–B). While MBC clearly increased with soil C, the allometric slope (0.88, Table 1) of this relationship falls below the 1∶1 line (isometric ratio based on geometric mean), indicating diminishing growth return of MBC with increasing soil C, which also appeared in forest and pasture soils (Table S1). These findings might suggest the availability of nutrients or labile C may limit biomass growth at higher soil C [7]. In contrast, we found that soil MBC pools were linearly related to soil N pools (allometric slope 0.99, Table 1, Fig. 4B) consistently across soil types, which could suggest that N availability more directly constrains the size of microbial C pools across global soils.

Comparison of soil and microbial stoichiometry may further suggest potential N limitation of microbial biomass pools, as the average N availability in soils (mean C∶N = 19∶1) is about half of that of microbial demand (mean C∶N = 11∶1, Table 1, Fig. 1). Moreover, microbial demand for N appears to generally increase in a size dependent manner with biomass C (and soil C pools), while soil N availability may slightly decline with soil C accumulation, and is particularly deficient in litter layers (Fig. 1A, Fig. S1A). Nitrogen limitation of soil microbial biomass pools been predicted by multiple element limitation models [61], and would appear consistent with the growth rate hypothesis (GRH), which links biomass growth to the synthesis of N rich proteins [16], [17].

While our results may appear to suggest soil microbes might be more limited by N than by C availability, this interpretation is challenged by empirical findings showing close association between microbial C and N acquisition. Soil N may be tightly bound with recalcitrant humic C in soils, and N mineralization may be dependent on enzymatic mining of recalcitrant soil C [62], [63]. As a result, addition of inorganic N to soils may decrease decomposition and soil respiration rates [63], [64], thought to be associated with suppression of enzymes breaking down humic C [7]. Although enzymatic models suggest that microbial biomass may increase in response to inorganic N additions [61], empirical data indicates soil microbial biomass may consistently decrease with N addition [65] potentially as the result of shifts in microbial community composition [66], [67]. These findings may ultimately support the hypothesis that microbial N mining drives decomposition of recalcitrant C in soils, and we posit that these responses to inorganic N additions may reflect preferential demand for N rather than C in soil microbial communities under ambient conditions where N is bound with humic C.

At global scales, soil microbial biomass C pools were not closely related to ecosystem P pools (Fig. 4C) or relative P availability. While microbial biomass was somewhat correlated with soil C∶P and N∶P ratios (Table S3), this may likely reflect covariation of C∶P and N∶P element ratios with soil C (Fig. S1), which was in turn more closely related to MBC (Table S3). Although a weak general relationship between soil total P and MBC could be described for pastures and forest soils (Fig. 4C, Table S1), MBC appeared decoupled from soil P pools at the extremes of soil C and MBC, which were lowest in crop soils, and highest in organic wetland soils, boreal forests, and litter and humus layers, despite similar concentrations of soil P (Fig. 4C). In contrast to tightly linked pools of C and N in soils and microbes, decoupling of microbial growth from soil P pools may present stark contrasts in microbial P demand relative to soil total P pools at the extremes of soil C accumulation (Fig. 1B–C, Fig. 4C). Soil microbial communities may have evolved several mechanisms to cope with these large variations in the relative availability of P across terrestrial habitats, including exudation of phosphatase enzymes, differential rates of P uptake and cycling [7], [9], [68], and stoichiometric variation (Fig. 2–3).

### Factors shaping microbial metabolic quotients

**Figure 5:**
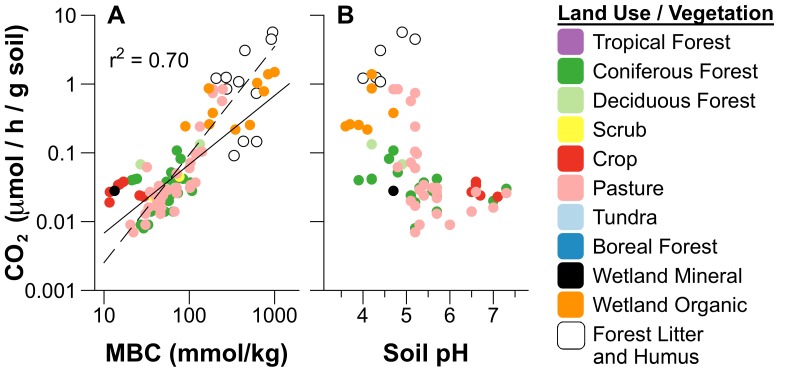
Carbon mineralization rates (CO_2_) varied with A) microbial biomass C (MBC) and B) soil pH. C mineralization rates were measured in glass jar incubations in studies with concurrent measurements of microbial biomass C, N, and P. Dashed line in A) is the regression fit, and solid line is the 1∶1 isometric line based on the geometric mean ratio of CO_2_ to MBC (mean *q*CO_2_). Parameters estimated by SMA are given in Table 1. SMA regressions fit using only forest and pasture soils (parameters in Table S1) are not shown as they were essentially the same as the global relationships.

Microbial turnover of soil carbon pools may vary as a factor of both the size of biomass pools of soil microbes, and their rate of metabolism per unit biomass. Soil carbon mineralization rates in controlled laboratory incubations were closely linked with the size of microbial biomass C pools (Fig. 5A), although we found increases in soil C mineralization with microbial biomass were not linear (Table 1). This non-linear relationship between CO_2_ and MBC was essentially the same when comparing all global soils (including litter—Table 1) to results obtained only from forest and pasture soils (Table S1). The allometric slope of the relationship between CO_2_ and MBC lies above the 1∶1 line of constant proportions (slope = 1.57, Table 1), indicating an exponential growth of C mineralization with MBC pool size. This exponential relationship between CO_2_ flux and microbial biomass C pools indicates the rate of metabolism *per unit biomass* increased with total biomass of soil microbes in laboratory incubations.

To assess controls on microbial metabolic rates, we determined the microbial metabolic quotient *q*CO*_2_* [29], essentially an analysis of the residual variation in CO_2_ flux after accounting for differences in soil microbial biomass (*q*CO_2_ = CO_2_/MBC). We acknowledge that the metabolic quotient *q*CO_2_ is an imperfect proxy for microbial growth rates, which does not describe biomass accumulation rates (δ MBC) or potential variation in the proportion of C incorporated into biomass compared to CO_2_ respired [6], [26], [69]. Although *q*CO_2_ may be interpreted as reflecting microbial C use efficiency (CUE) [70], derived from the proportion of C incorporated into biomass : C respired (CUE = δ MBC/(δ MBC+δ CO_2_)) [71], [72], soil microbial growth rates are difficult to measure, and without δ MBC data, *q*CO_2_ is not an appropriate metric of CUE [72]. Given the lack of data on MBC accumulation rates in soils, we view *q*CO_2_ as a readily determined index of microbial metabolic rates (respiration *per unit biomass*), for which existing data can be used to explore empirical support for the GRH in soil microbes.

**Figure 6:**
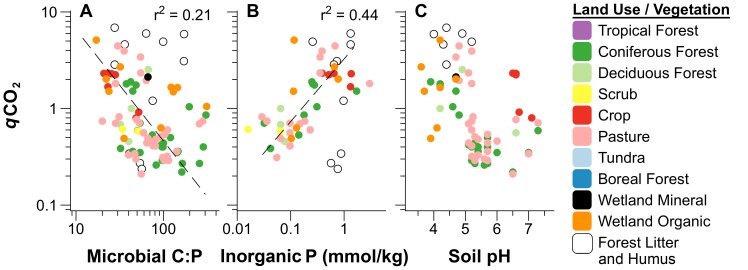
Factors influencing microbial metabolic quotients (*q*CO_2_) of soil incubations. Significant factors included A) microbial C∶P ratios, B) available inorganic P, and C) soil pH. *q*CO_2_ was calculated as the mol/mol ratio of C mineralization rates measured in glass jar incubations per unit microbial biomass C obtained from the same soils, with units of mmol CO_2_-C/h/mol MBC-C/g soil. Relationships of *q*CO_2_ with microbial C∶P (A), and inorganic P (B) were fit without data from litter or soil humic horizons. Data were log_10_ transformed for normality and parameters estimated by SMA are given in Table 1. SMA regressions fit using only forest and pasture soils (parameters in Table S1) are not shown as they were essentially the same as the global relationships.

The growth rate hypothesis would suggest microbial metabolic rates should be closely coupled with biomass C∶P ratios [16], [17], and we found a weak but significant negative relationship between microbial C∶P ratios and *q*CO_2_ in existing data from soil incubations (Fig. 6A). The negative sign of the regression relationship between microbial biomass C∶P ratios and *q*CO_2_ indicates higher rates of metabolism (*q*CO_2_) corresponded with greater microbial P concentrations (lower C∶P), as the GRH would suggest (Fig. 6A, Table 1, Table S1). We also tested multivariate regression models of CO_2_ and *q*CO_2_, and found that although microbial C∶P ratios were a persistent factor in the most robust models, microbial C∶P only predicted soil *q*CO_2_ strongly in combination with additional factors including soil stoichiometry, pH, vegetation and climate (Tables S13, S14).

We found that soil available inorganic phosphorus (P_i_) was the single strongest predictor of microbial metabolic quotients (*q*CO_2_) in soil incubations from a wide range of terrestrial ecosystems spanning deserts, crop lands, pastures, coniferous and deciduous forests, and organic wetland soils (Fig. 6B). Addition of inorganic P to soils has been shown to accelerate decomposition rates in temperate forest soils [2], and differences in inorganic P concentrations were recently found to describe variation in rates of C mineralization among land use types including crops, pastures, and pine and hardwood forests [28]. However, to our knowledge the present work is the first to show shifts in soil microbial metabolic quotients (*q*CO_2_) with inorganic P availability. This result may suggest that the effects of soil inorganic P on C cycling recently observed by others [2], [6], [28] could arise from changes in mass-specific rates of microbial metabolism with P availability (Fig. 6B) rather than shifts in microbial biomass, which appeared to be limited by N and C pools in soils (Fig. 4A–B).

Our results also showed soil pH strongly affected both rates of carbon mineralization (CO_2_) and microbial metabolism (*q*CO_2_) across terrestrial soils, with non-linear changes in respiration and metabolism appearing to shift about a pH value of 5.5 (Figs. 5B, 6C). Variation in soil *q*CO_2_ with soil pH has been described in previous studies as the result of increased of maintenance respiration required for metabolic responses to pH stress [29], [73]. However, shifts in microbial metabolism with pH might also be related to changes in soil microbial communities, as both fungal∶bacterial ratios [74], [75] and the taxonomic composition of soil bacteria vary with pH across a wide range of soils, and may particularly shift about a soil pH value near 5.5 [57], [58], [76], [77], [78], similar to patterns we observed in *q*CO_2_. Importantly, the relative abundance of some bacterial taxonomic groups appear to be linked with soil respiration rates, suggesting r - vs. K - selection of microbial life strategies based on differences in growth rates [79].

### Is there a Biological Stoichiometry of the soil microbial biomass?

The growth rate hypothesis (GRH) has been shown to link the elemental composition of organisms to their metabolic rates in higher autotrophs and heterotrophs, with important implications for trait differentiation, community structure, and element cycling in terrestrial and aquatic ecosystems [11], [16], [18], [19], [20], [21], [22], [23], [24]. Similarly, relationships between bacterial metabolism, RNA content, and biomass C∶P supporting the GRH have been observed in cultured bacterial strains and mixed aquatic assemblages [14], 18,80. Importantly, both biomass stoichiometry and the degree of homeostatic response to nutrient availability appear to vary among different species of cultured bacteria [13], [14], [80], and with the degree of nutrient limitation [15], [42]. In culture and aquatic microcosms, mixed species assemblages may facilitate greater stoichiometric flexibility and less resource homeostasis at the community level, presumably as the result of shifts in community composition [14], [41], [42], [60]. However, different nutrient use strategies may coexist due to niche partitioning in mixed communities, potentially driving nutrient co-limitation (and apparent homeostasis) of mixed assemblages [41], [42].

We postulate that similar processes might govern the stoichiometry of microbial communities in soils, which have lower relative P availability and C lability than aquatic habitats [81]. While we acknowledge the potential for measurement error in soil microbial biomass pools, we observed considerable variation in microbial C∶N (up to one order of magnitude), and especially C∶P and N∶P ratios (up to two orders of magnitude) across global ecosystems (Fig. S3). This stoichiometric variaton was comparable to biological variation in bacterial cultures and aquatic microcosms [15], [38], [42], which also show greater flexibility in biomass C∶P and N∶P ratios than C∶N ratios [15], [38], [82], [83].

However, our results showed microbial stoichiometry largely did not appear responsive to resource ratios in soils [10], unlike non-homeostatic patterns often found in microbes in culture and aquatic microcosms [15], [41], [42], [60]. This apparent resource homeostasis might be related to community level observations inherent in analysis of soil microbes [25], and the potential for niche partitioning to drive community homeostasis [41], [42]. However, cultured and aquatic microbes may show strict homeostasis only under P limitation [41], [42], [82], and the homeostatic responses of soil microbes could conceivably be viewed as reflecting widespread limitation by soil P availablilty (Fig. S1).

Although we found only limited evidence that microbial stoichiometry was not homeostatic with respect to soil resources (Tables S11, S12), several of our findings suggest soil microbes are not Strictly Homeostatic (i.e. fixed, isometric stoichiometry [37]). Our results showed patterns of size-dependent scaling and habitat specific variation in soil microbial stoichiometry closely analogous to those in cultured and aquatic microbes [12], [14], [15], [38], [42], [60]. These size-dependent and habitat specifc differences in microbial stoichiometry may have important implications for ecosystem element retention and fluxes [38], [42], including microbial turnover of nutrients and C in soils and litter [6], [25], [26]. Concommitant changes in microbial stoichiometry and metabolism along land use gradients [44], [45], [47], [84], and dynamic variation in soil microbial stoichiometry with season, soil moisture [51], [52], [53], [54], [55], and wetland innundation [85], [86], [87], could also suggest variation in biomass stoichiometry is linked with shifts in soil microbial activity.

We found microbial stoichiometry and metabolic rates appeared directly linked in soils, with a weak but significant relationship between microbial biomass C∶P ratios and metabolic quotients (*q*CO_2_) across a wide range of global soils. This finding parallels the association between rates of cellular growth and biomass C∶P ratios described by the GRH [16], [17], although the analogy is imperfect as our observations represent mixed communites rather than individual organisms, and we used a crude index of microbial metabolism (*q*CO_2_) as a proxy for growth rates.

We also found that microbial metabolic quotients (*q*CO_2_) were most strongly associated with inorganic P availability across global soils, consistent with increased rates of C cycling associated with P availability and P fertilization, even in soils considered to be N limited [2], [28]. In contrast, our results suggested that soil microbial biomass pools appeared constrained primarily by soil N. Although inorganic N additions to soil appear inconsistent with our results, we contend these manipulations may critically alter ecosystem processes and soil communities relative to ambient conditions [63], [64], [66], [67].

We suggest differential nutrient limitation of microbial biomass and metabolism across terrestrial and wetland soils may broadly reflect biochemical mechanisms described by the GRH, where relationships between growth rates and organismal stoichiometry arise from the control of P-rich ribosomes and rRNA pools over rates of synthesis of N-rich proteins [16], [17], This mechanism essentially implies N limitation of the structural components of cells (biomass) and P limitation of their metabolic rates [16], [17], and our analogous findings in soil microbes may implicate these basic processes of cellular metabolism in the cycling and retention of soil nutrients and C at global scales.

Our findings suggest further study is needed to investigate potential linkages between microbial stoichiometry, metabolism, and community composition [25], [80] in soils. We note that the primary controls over microbial community composition across terrestrial and wetland soils are land use and pH [57], [58], [59], [76], [78], which were associated with respective shifts in microbial stoichiometry and metabolism in our study. We observed non-linear patterns in inflection points of soil CO_2_ flux and metabolic quotients near soil pH 5.5, a value strikingly similar to widely observed change points in soil microbial community composition and diversity. Specific groups of soil bacteria have been linked to variation in soil C mineralization rates across terrestrial soils, which may indicate an energetic basis of ecological strategies in soil microbes [79] that also appears to describe microbial community responses to soil N addition [67], [88]. The ecological strategies of soil bacteria may also appear directly related to differences in their ribosome copy number [56], [89], suggesting GRH-like mechanisms could differentiate functional groups and shape microbial community structure and function in soils.

## Materials and Methods

To determine the influence of resource availability on microbial growth and metabolism in soils, we compiled data on soil and microbial stoichiometry and respiration, and metabolism from existing published studies, focusing exclusively on studies including measurements of microbial C, N, and P pools in soils. Our approach to literature review and data collection was broadly similar to that of Cleveland and Lipzin [10], although we used a different search approach to obtain a greater number of publications encompassing a broader range of soil habitats (including wetlands and crops), and explicitly categorized the major habitats, vegetation types, and land uses from which samples were obtained. Importantly, we collected additional data parameters not included in the previous study [10], particularly measurements of soil C mineralization and *q*CO_2_, along with measurements of available inorganic P and soil pH where data was available in studies simultaneously measuring microbial biomass C,N, and P pools.

### Literature Review

To efficiently obtain publications with complete microbial biomass C, N, and P data, all literature searches included the term “microbial biomass phosphorus” (including quotes), as studies of microbial biomass P (MBP) often include data on microbial C and N pools, while more prevalent studies of microbial C∶N stoichiometry often do not analyze microbial P [10]. Rather than use a citation based literature search based upon common methods for determining microbial P pools [10], we used a general search for “microbial biomass phosphorus” to obtain publications that might cite the authors' earlier work rather than the original methods papers. Literature searches were conducted with Google Scholar, and search results were compiled in bulk using the Zotero plug-in (www.Zotero.org) for the Firefox browser [90]. Redundant citations were removed from the search results database and publications were screened based on the inclusion of data on microbial biomass P and other microbial biomass pools.

To obtain a dataset with the most comprehensive coverage of global terrestrial ecosystems, we conducted a stratified literature search, with general searches followed by searches for specific ecosystem types. All searches contained the text “microbial biomass phosphorus,” with additional search strings describing terrestrial biomes and land uses along with wetland habitats. A general search was conducted using the string ““microbial biomass phosphorus,” “C/P”, and “soil””. In place of “soil” additional stratified searches were used to capture results in different ecosystem types (11 terms, e.g. “tropical”, “desert”, “boreal,” etc.) and wetland habitats (9 terms, e.g. “wetland”, “peatland”, “bog”, “fen”, “salt marsh”, “floodplain,” etc.). However, these additional terms yielded no unique results beyond the general search using “soils”. To find studies measuring both MBP and soil C cycling, the search ““microbial biomass phosphorus,” and “C/P””, was repeated with additional terms “CO2” and “qCO2” in separate iterations.

### Data collection and processing

Our search results yielded 238 unique journal articles, from which we obtained 107 candidate publications with potentially relevant data on microbial biomass element pools. However, fewer studies (66) were used to obtain the final data set due to the exclusion of studies lacking required data layers, or with data presented in unusable formats, or from non-soils or unusually disturbed soils (Fig. S5). Publications used as data sources are listed in (Table S15), and citations for these data references are provided as Text S1. Data was obtained only from publications measuring microbial biomass C, N, and P concentrations using standard chloroform fumigation-extraction methods (0.5 M KCl to extract biomass C and N, and 0.5 M NaHCO_3_ to extract biomass P) following [10]. In contrast to the prior study [10], we obtained data from as many soils as possible, including soil samples representing altered human land use regimes (e.g. crops, pastures and grazed savannas). We further cross-classified soils and soil datasets by climate, land use, and vegetation (Tables S16, S17).

Our final dataset included measurements from 355 soils published in 66 studies (Table S18), a substantial increase from the prior study [10], which obtained 186 soils from 48 studies. To standardize data and allow for comparison of elemental stoichiometry, all measurements of microbial biomass C, N, and P were converted into units of mmol/kg soil. Where data presented were not calculated using conversions for extraction efficiency, standard correction coefficients (0.45 for microbial C and N, 0.40 for microbial P) were applied [91]. Data were also collected for total pools of soil C, N, and P, and converted to mmol/kg soil. Extractable inorganic phosphorus data was obtained from studies using the Olsen extraction (0.5 M NaHCO_3_), which is thought to reflect soil P availability [31], and data was converted to mmol P/kg soil. However, soil extractable inorganic N was not included in our dataset, as few studies included this variable. Soil pH data was collected where available, as soil pH is known to strongly affect the community composition of soil microbes [57], [78].

We also collected available data on soil C mineralization rates where controlled experiments were conducted concurrently with measurements of microbial biomass C, N, and P pools in soils. Soil C mineralization data were only collected from studies that determined respiration rates in jars using soil and litter incubations with standardized moisture and temperature, with the average hourly respiration over a ten hour period [92] used to obtain an average rate of C mineralization expressed as mmol CO_2_-C/h/g soil. Metabolic rates of soil microbial communities were indexed by calculating the metabolic quotient *q*CO_2_ [29], [92], by dividing C mineralization rates (per g soil) by microbial biomass (per g soil), to yield *q*CO_2_ values expressed in mmol CO_2_-C/mol MBC/h.

### Statistical methods

Nutrient concentrations in the environment are often distributed log-normally [93], and measurements of element pools, fluxes, and ratios in soils were log_10_ transformed to improve normality. Stoichiometric relationships in soils and microbes were determined using a size-dependent approach, which describes allometric relationships based on the power function *y = a x^b^* [10], [11]. Log transformation of this power relationship yields the linear function *log y = a+b (log x)*, allowing the use of linear regressions of log_10_ transformed data to determine stoichiometric relationships among nutrients in soils and microbes. Bivariate relationships between soil and microbial element pools, and ecosystem C fluxes were determined using standardized major axis (Type II) regression using the SMATR 3.0 package [94] in the open source *R* statistical software program (www.r-project.org), which we used to test whether slopes of allometric relationships were isometric (Slope = 1). Results of SMA regressions of each pairwise combination of all variables in our dataset are given in (Tables S3, S4).

Variation in the stoichiometry of soils and microbes, along with soil CO_2_ flux and metabolic quotients (*q*CO_2_) were compared among climate regions and vegetation by one-way ANOVA, and interactions with allometric slopes were tested using SMATR. Pairwise differences among habitat types were determined by Tukey's tests, and general linear models were used to determine the proportion of variance in soils, microbes, and metabolism explained by climate and vegetation groups. We also compared multivariate general linear models of factors influencing soil CO_2_ flux and *q*CO_2_, using an exhaustive search of all combinations of factors in our dataset (Tables S13, S14).

## Supporting Information

Figure S1 **Scaling of soil stoichiometric ratios with soil carbon (C) accumulation.** Stoichiometric variation of A) soil C∶N, B) C∶P, and C) N∶P ratios are shown as a function of soil C, and soil stoichiometry and C data were log_10_ transformed for normality. Dashed lines indicate SMA regression fits, and solid lines indicate the Redfield (1958) ratios (C∶N∶P = 106∶16∶1), while dotted horizontal lines show the average C∶N∶P ratios of soil microbes in this study (Table 1). Soil C∶N (A) was fit separately for organic soil horizons and forest litter, and for all other soils. Litter and organic soils did not show relationships with C∶P and N∶P ratios as a function of soil C. All SMA regression parameters are given in Table S2.
(TIF)

Figure S2 **Contribution of soil C∶N and C∶P ratios to variation in N∶P ratios across global soils.** Relationships of soil N∶P with A) soil C∶N and B) soil C∶P were determined seperately for only litter and humic horizons, and for all other soils exclusive of forest litter and humus. SMA fits for litter and humic horizons are shown with dotted black lines with correlation coefficients in italics. Dashed black lines show SMA fits for all soils less litter and humic horizons, with correlation coefficients in plain text. Soil stoichiometric data were log_10_ transformed for normality, and parameter estimates for SMA regressions are given in Table S2.
(TIF)

Figure S3 **Comparisons of microbial biomass C∶N∶P stoichiometric ratios with corresponding soil C∶N∶P stoichiometry.** Relationships between microbial and soil stoichiometry are shown for A) C∶N ratios, B) C∶P ratios, and C) N∶P ratios. Soil and microbial stoichiometry were log_10_ transformed for normality. No regression fits are shown as none of these relationships were significant (Table S2).
(TIF)

Figure S4 **Stoichiometric scaling of microbial biomass C, N, and P by land use and vegetation categories.** Data and SMA regression fit lines are shown for each land use and vegetation category to express habitat level differences in scaling of microbial biomass A) C∶N ratios, B) C∶P ratios, and C) N∶P ratios. Microbial biomass element pool data were log_10_ transformed for normality prior to fitting SMA regressions for each treatment simultaneously, and treatments without significant fits are not shown. Open circles indicate data where regressions were not individually significant, while significant relationships are plotted with data as solid points. Regression fits are shown with colored lines, with red solid lines indicating slopes were not equal to 1, and blue lines showing slopes not significantly different from 1. Thin black dotted lines show the regression fits for all groups combined (the same as in Fig. 1), while thin black solid lines indicate the Redfield (1958) ratios (C∶N∶P = 106∶16∶1). Parameters estimated by SMA regressions are provided in Tables S5, S6, S7, along with results of intercept and slope tests and multiple comparisons of these parameters.
(TIF)

Figure S5 **Flow of included studies for used as data sources for the meta-analysis.**
(PDF)

Table S1 **Summary of SMA regressions of log_10_-transformed C, N, and P contents in soil and microbial pools, along with predictors of soil C mineralization (CO_2_) and microbial metabolism (** ***q*** **CO_2_), considering only data from forest and pasture soils.** Bivariate relationships were significant (P<0.001) for all relationships shown. Slopes significantly different from one (P>0.05) are shown in boldface font. Slopes not different from one (not bold) indicate an isometric (linear) relationship among parameters. The geometric mean and standard errors (SE) of stoichiometric ratios (x∶y ratio, x∶y mean) are given for reference, but are not representative where allometric slopes different from 1. The coefficient of variation (CV) of stoichiometric ratios is given as a dimensionless index of dispersion about the mean. Single asterisks (*) indicate where different slopes are observed by considering only forest and pasture soils compared to the full range of sites (presented in Table 1), and ** indicates a different relationship was tested for only litter and organic soils (wetland organic, boreal forest, and humic horizons).
(DOCX)

Table S2 **Summary of SMA regressions of log_10_-transformed relationships among soil C and soil stoichiometry, paired analysis of soil stoichiometric ratios, and comparisons of soil and microbial stoichiometry.** SMA regression fits for these relationships correspond with data presented in Figs. S1, S2, S3. Bivariate relationships were significant (P<0.001) for all relationships shown., unless otherwise noted (n.s.). Slopes significantly different from one (P>0.05) are shown in boldface font. Slopes not different from one (not bold) indicate an isometric (linear) relationship among parameters. Analysis of relationships between soil C∶N ratios and soil C accumulation were divided by habitat given different relationships observed among different vegetation types (Fig. S1). These groupings corresponded with soil C content greater than 24% C (20,000 mmol/kg) for habitats including boreal forests, wetland organic soils, and litter, and soil C content less than 24% (all other habitats).
(DOCX)

Table S3 **Results of all pairwise SMA regressions among log_10_-transformed study variables including soil and microbial C, N, and P pools, and soil stoichiometric ratios.** Microbial biomass C, N, and P are abbreviated MBC, MBN, and MBP. P_i_ is the concentration of extractable inorganic (Olsen) P (available P), and P_i_∶P is the ratio of inorganic P to soil total P. Relationships among variables were compared using Standardized Major Axis (Type II) regression (SMA), except for relationships with habitat categories, which were assessed using generalized linear models (GLM). Only relationships with P>0.05 and r^2^ (SMA) or R^2^ (GLM)>0.25 are shown for clarity, except where data are displayed graphically in separate figures (boldface). Italics indicate relationships that are autocorrelated by their definition.
(DOCX)

Table S4 **Results of all pairwise SMA regressions among log_10_-transformed study variables including soil microbial stoichiometry, respiration, and metabolism (** ***q*** **CO_2_).** Microbial biomass C∶N, C∶P, and N∶P ratios are abbreviated by mC∶N, mC∶P, and mN∶P, respectively. The ratio of microbial P to soil total P is abbreviated P_m_∶P, and P_m_∶P_i_ is the ratio of microbial P to inorganic P. Relationships among variables were compared using Standardized Major Axis (Type II) regression (SMA), except for relationships with habitat categories, which were assessed using generalized linear models (GLM). Only relationships with P>0.05 and r^2^ (SMA) or R^2^ (GLM)>0.25 are shown for clarity, except where data are displayed graphically in separate figures (boldface). Italics indicate relationships that are autocorrelated by their definition.
(DOCX)

Table S5 **SMA parameter estimates for simultaneous fitting of microbial biomass C and N scaling relationships by land use and vegetation categories.** Regression fit lines are compared by category in Fig. 3A, and data and regression fits are plotted by category in Fig. S4A. The simultaneous SMA relationships for microbial biomass C and N scaling were tested for differences in intercepts (P<0.001) and slopes (P<0.001), and significantly different intercept and slope groups were determined by multiple comparisons in SMATR v.3.0, controlling the overall error rate at p<0.05. Slopes of individual relationships significantly different from one are shown in boldface. For each category, geometric mean of C∶N ratios are presented (± SE) with their coefficient of variation (CV), and grouping by multiple comparisons using Tukey's test (p<0.05) on log_10_-transformed data.
(DOCX)

Table S6 **SMA parameter estimates for simultaneous fitting of microbial biomass C and P scaling relationships by land use and vegetation categories.** Regression lines are compared by category in Fig. 3B, and data and regression fits are plotted by category in Fig. S4B. The simultaneous SMA relationships for microbial biomass C and P scaling were tested for differences in intercepts (P<0.001) and slopes (P = 0.284), and significantly different intercept and slope groups were determined by multiple comparisons in SMATR v.3.0, controlling the overall error rate at p<0.05. Slopes of individual relationships significantly different from one are shown in boldface. For each category, geometric mean of C∶P ratios are presented (± SE) with their coefficient of variation (CV), and grouping by multiple comparisons using Tukey's test (p<0.05) on log_10_-transformed data.
(DOCX)

Table S7 **SMA parameter estimates for simultaneous fitting of microbial biomass N and P scaling relationships by land use and vegetation categories.** Regression lines are compared by category in Fig. 3C, and data and regression fits are plotted by category in Fig. S4C. The simultaneous SMA relationships for microbial biomass N and P scaling were tested for differences in intercepts (P<0.001) and slopes (P = 0.012), and significantly different intercept and slope groups were determined by multiple comparisons in SMATR v.3.0, controlling the overall error rate at p<0.05. Slopes of individual relationships significantly different from one are shown in boldface. For each category, geometric mean of N∶P ratios are presented (± SE) with their coefficient of variation (CV), and grouping by multiple comparisons using Tukey's test (p<0.05) on log_10_-transformed data.
(DOCX)

Table S8 **SMA parameter estimates for simultaneous fitting of microbial biomass C and N scaling relationships by climate categories.** The simultaneous SMA relationships were tested for differences in intercepts (P<0.001) and slopes (P<0.001), and significantly different intercept and slope groups were determined by multiple comparisons in SMATR v.3.0, by controlling the overall error rate at p<0.05. Bivariate relationships of log_10_-transformed data were significant (P<0.001) for all relationships shown, unless otherwise noted due to insufficent data. Slopes significantly different from one (P>0.05) are shown in boldface font. For each category, geometric mean of N∶P ratios are presented (± SE) with their coefficient of variation (CV), and with grouping by multiple comparisons using Tukey's test (p<0.05) on log_10_-transformed data.
(DOCX)

Table S9 **SMA parameter estimates for simultaneous fitting of microbial biomass C and P scaling relationships by climate categories.** The simultaneous SMA relationships were tested for differences in intercepts (P<0.001) and slopes (P<0.001), and significantly different intercept and slope groups were determined by multiple comparisons in SMATR v.3.0, by controlling the overall error rate at p<0.05. Bivariate relationships of log_10_-transformed data were significant (P<0.001) for all relationships shown, unless otherwise noted due to insufficent data. Slopes significantly different from one (P>0.05) are shown in boldface font. For each category, geometric mean of N∶P ratios are presented (± SE) with their coefficient of variation (CV), and with grouping by multiple comparisons using Tukey's test (p<0.05) on log_10_-transformed data.
(DOCX)

Table S10 **SMA parameter estimates for simultaneous fitting of microbial biomass N and P scaling relationships by climate categories.** The simultaneous SMA relationships were tested for differences in intercepts (P<0.001) and slopes (P<0.001), and significantly different intercept and slope groups were determined by multiple comparisons in SMATR v.3.0, by controlling the overall error rate at p<0.05. Bivariate relationships of log_10_-transformed data were significant (P<0.001) for all relationships shown. Slopes significantly different from one (P>0.05) are shown in boldface font. For each category, geometric mean of N∶P ratios are presented (± SE) with their coefficient of variation (CV), and with grouping by multiple comparisons using Tukey's test (p<0.05) on log_10_-transformed data.
(DOCX)

Table S11 **SMA parameter estimates for simultaneous fitting of homeostatic relationships between microbial and soil stoichiometry by vegetation categories.** The simultaneous SMA relationships were tested for differences in intercepts (P<0.001) and slopes (P<0.001). Slopes significantly different from one (P>0.05) are shown in boldface font. Only significant relationships with r^2^≥0.3 and n>5 are shown.
(DOCX)

Table S12 **SMA parameter estimates for simultaneous fitting of homeostatic relationships between microbial and soil stoichiometry by climate categories.** Microbial biomass C∶N, C∶P, and N∶P ratios are abbreviated by mC∶N, mC∶P, and mN∶P, respectively. The simultaneous SMA relationships were tested for differences in intercepts (P<0.001) and slopes (P<0.001). Slopes significantly different from one (P>0.05) are shown in boldface font. Only significant relationships with r^2^≥0.3 and n>5 are shown.
(DOCX)

Table S13 **Multivariate general linear regression models of soil C mineralization rates (CO_2_) as a function of ecosystem, soil and microbial factors.** General linear models were compared using an exhaustive search, but only selected models with all predictors simultaneously significant are shown. To account for differences in missing data among parameters, we computed the test statistic %Var = R^2^ * (n samples in model/n total samples). Model 9 (bold) had the greatest %Var, explaining more variance in more data points. Notably, addition of soil pH to models (Models 10–14) improved fit (R^2^), but at the cost of fewer observations (df), resulting in a lower % Var. Models including both pH and inorganic P (P_i_) had far fewer observations (Models 15–19), and consequently lower % Var explained. Linear regression results for individual predictors of CO_2_ by Standardized Major Axis regression (SMA) are given in Table S4.
(DOCX)

Table S14 **Multivariate general linear regression models of the microbial metabolic quotient** ***qCO_2_*** **as a function of ecosystem, soil and microbial factors.** General linear models were compared using an exhaustive search, but only selected models with all predictors simultaneously significant are shown. To account for differences in missing data among parameters, we computed the test statistic %Var = R^2^ * (n samples in model/n total samples). Model 9 had the greatest %Var and lowest AIC. Linear regression results for individual predictors of *q*CO_2_ by Standardized Major Axis regression (SMA) are given in Table S4.
(DOCX)

Table S15 **Publications used as sources of soil and microbial element pool data.** Ref. no. refers to reference citation number for this article, with most data source publication references given in Text S1. Data Ref. no. indicates the numbering used in the extracted data set presented in Table S18.
(DOCX)

Table S16 **Codes for climate categories used to describe soils in the full microbial stoichiometry data set (Table S18).**
(DOCX)

Table S17 **Codes used to describe land use and vegetation classification of soils in the full microbial stoichiometry data set (Table S18).**
(DOCX)

Table S18 **Data set obtained for soil and microbial stoichiometry, and C mineralization across ecosystems, classified by climate (Clim) and land use (LU).** Codes for climate and land use categories are given in Tables S11 and S12, respectively. All soil chemical and microbial pools are expressed as mmol/kg soil. Soil respiration rate from standardized incubations (CO_2_) is expressed as µmol CO_2_-C/g soil/h.
(XLSX)

Text S1 **Additional references for publications used as data sources.**
(DOCX)
