# Supplementary figures and images for: Differential Nutrient Limitation of Soil Microbial Biomass and Metabolic Quotients (qCO2): Is There a Biological Stoichiometry of Soil Microbes?
Source: PLoS One. 2013 Mar 19;8(3):e57127. doi: 10.1371/journal.pone.0057127 (PMC3602520; doi:10.1371/journal.pone.0057127)

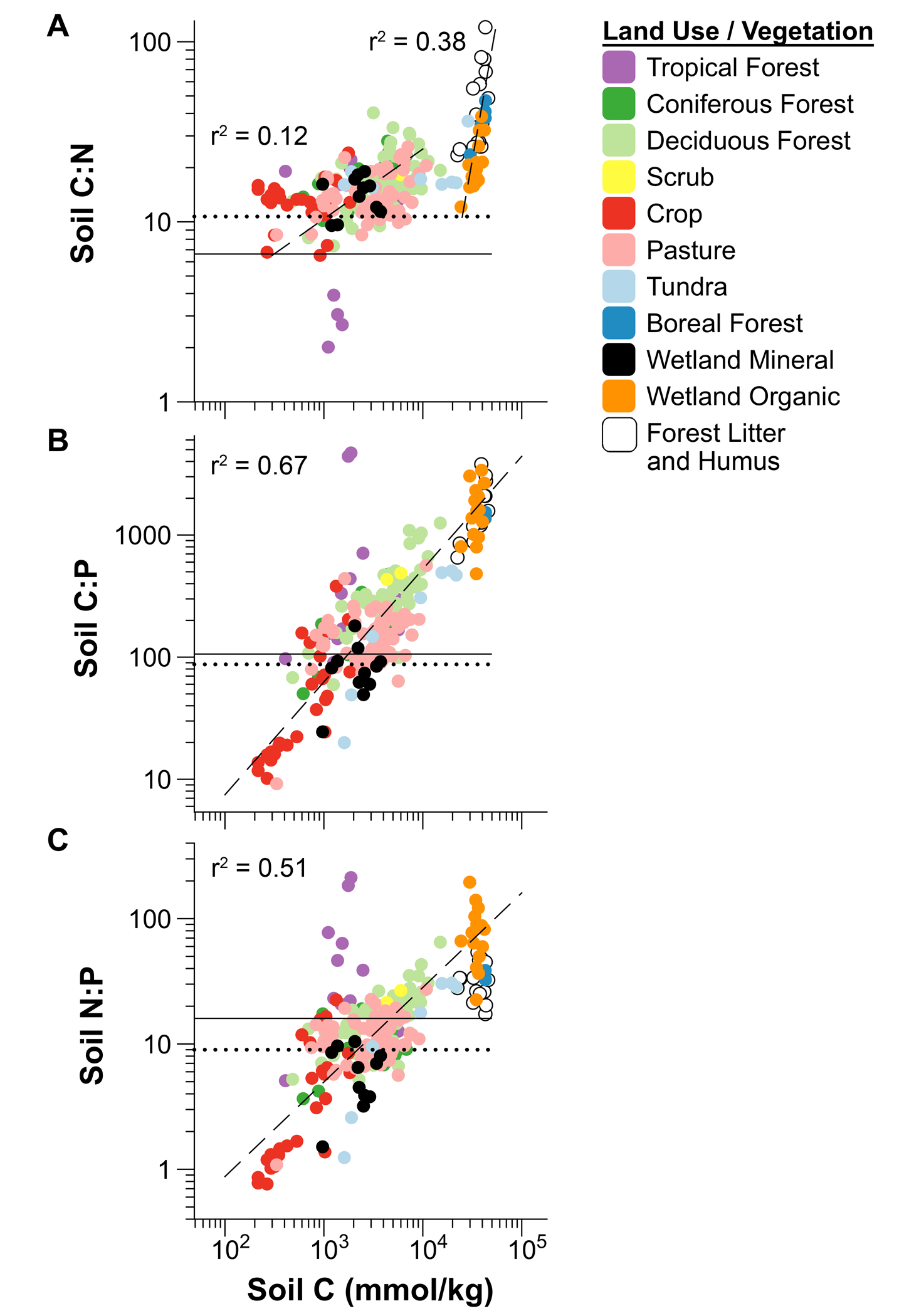

Supplement: Figure S1 — Scaling of soil stoichiometric ratios with soil carbon (C) accumulation. Stoichiometric variation of A) soil C∶N, B) C∶P, and C) N∶P ratios are shown as a function of soil C, and soil stoichiometry and C data were log10 transformed for normality. Dashed lines indicate SMA regression fits, and solid lines indicate the Redfield (1958) ratios (C∶N∶P = 106∶16∶1), while dotted horizontal lines show the average C∶N∶P ratios of soil microbes in this study (Table 1). Soil C∶N (A) was fit separately for organic soil horizons and forest litter, and for all other soils. Litter and organic soils did not show relationships with C∶P and N∶P ratios as a function of soil C. All SMA regression parameters are given in Table S2. (TIF) [file pone.0057127.s001.tif]

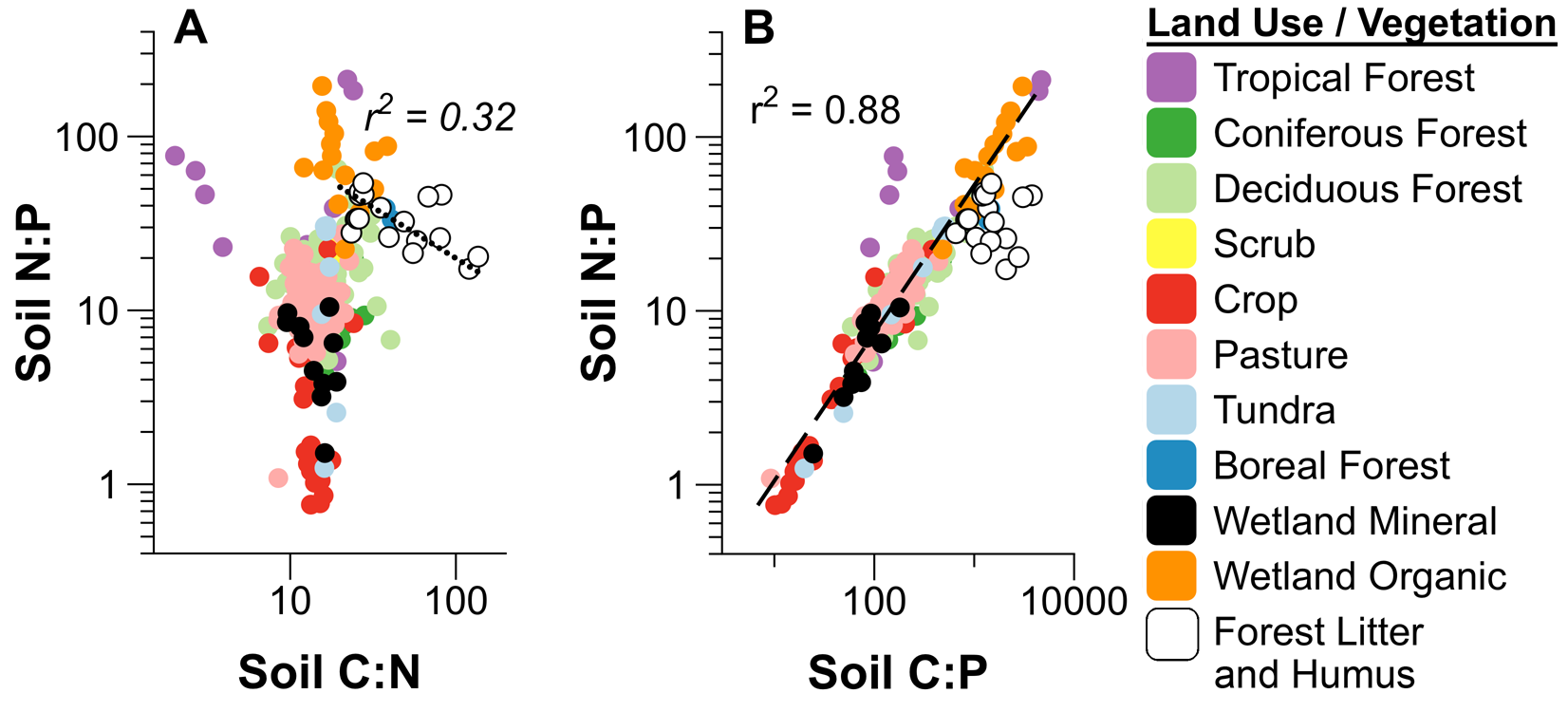

Supplement: Figure S2 — Contribution of soil C∶N and C∶P ratios to variation in N∶P ratios across global soils. Relationships of soil N∶P with A) soil C∶N and B) soil C∶P were determined seperately for only litter and humic horizons, and for all other soils exclusive of forest litter and humus. SMA fits for litter and humic horizons are shown with dotted black lines with correlation coefficients in italics. Dashed black lines show SMA fits for all soils less litter and humic horizons, with correlation coefficients in plain text. Soil stoichiometric data were log10 transformed for normality, and parameter estimates for SMA regressions are given in Table S2. (TIF) [file pone.0057127.s002.tif]

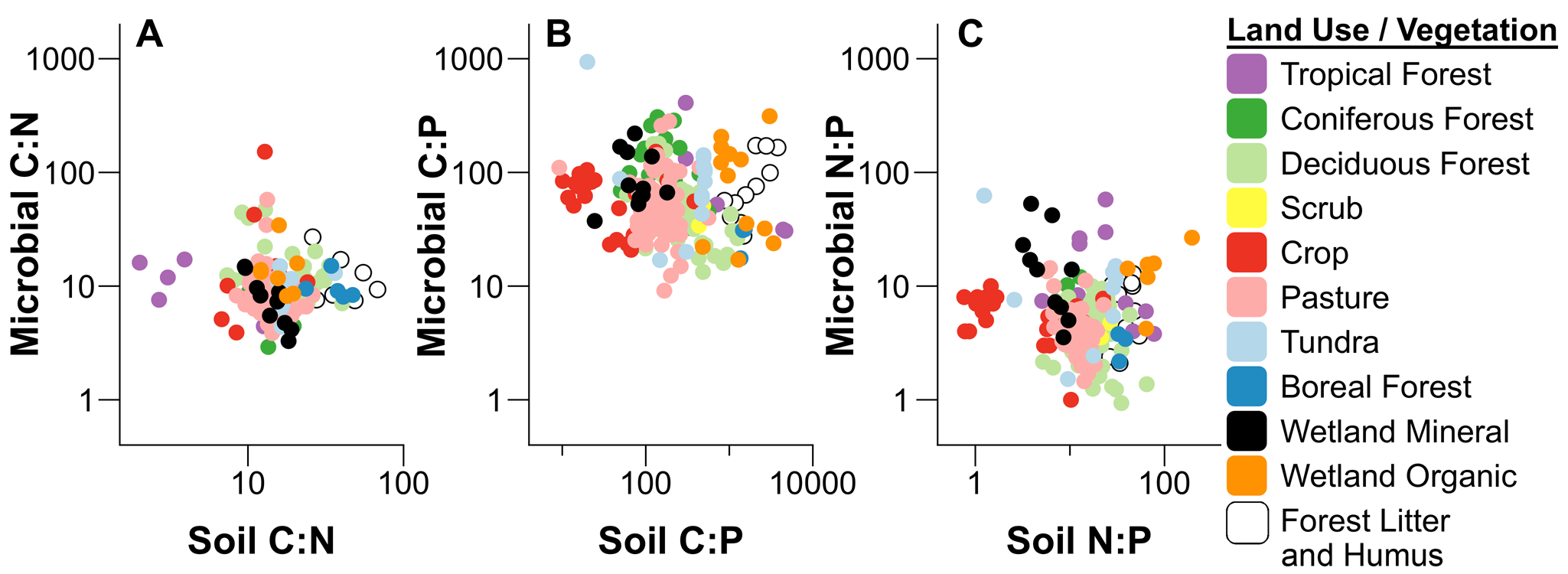

Supplement: Figure S3 — Comparisons of microbial biomass C∶N∶P stoichiometric ratios with corresponding soil C∶N∶P stoichiometry. Relationships between microbial and soil stoichiometry are shown for A) C∶N ratios, B) C∶P ratios, and C) N∶P ratios. Soil and microbial stoichiometry were log10 transformed for normality. No regression fits are shown as none of these relationships were significant (Table S2). (TIF) [file pone.0057127.s003.tif]

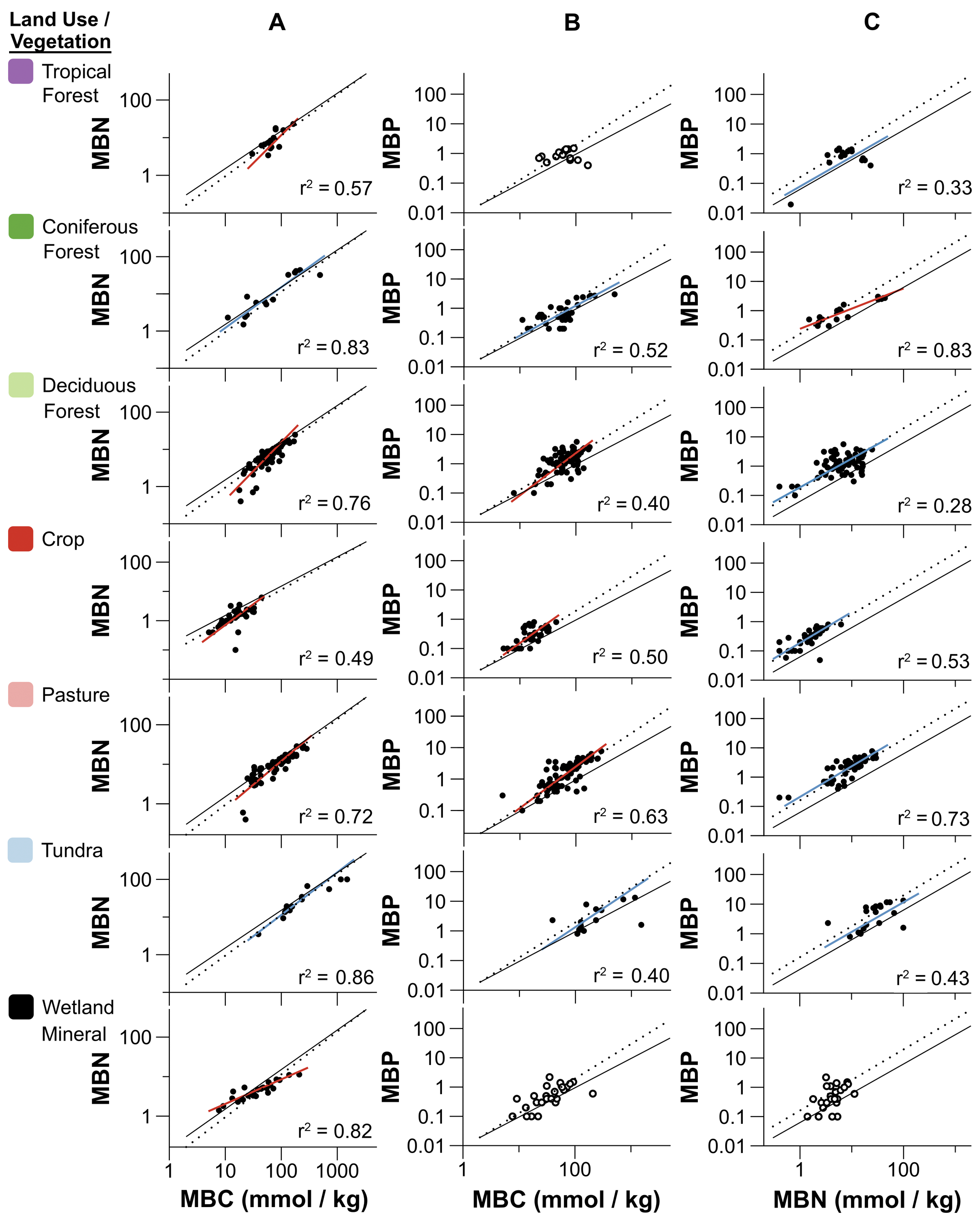

Supplement: Figure S4 — Stoichiometric scaling of microbial biomass C, N, and P by land use and vegetation categories. Data and SMA regression fit lines are shown for each land use and vegetation category to express habitat level differences in scaling of microbial biomass A) C∶N ratios, B) C∶P ratios, and C) N∶P ratios. Microbial biomass element pool data were log10 transformed for normality prior to fitting SMA regressions for each treatment simultaneously, and treatments without significant fits are not shown. Open circles indicate data where regressions were not individually significant, while significant relationships are plotted with data as solid points. Regression fits are shown with colored lines, with red solid lines indicating slopes were not equal to 1, and blue lines showing slopes not significantly different from 1. Thin black dotted lines show the regression fits for all groups combined (the same as in Fig. 1), while thin black solid lines indicate the Redfield (1958) ratios (C∶N∶P = 106∶16∶1). Parameters estimated by SMA regressions are provided in Tables S5, S6, S7, along with results of intercept and slope tests and multiple comparisons of these parameters. (TIF) [file pone.0057127.s004.tif]

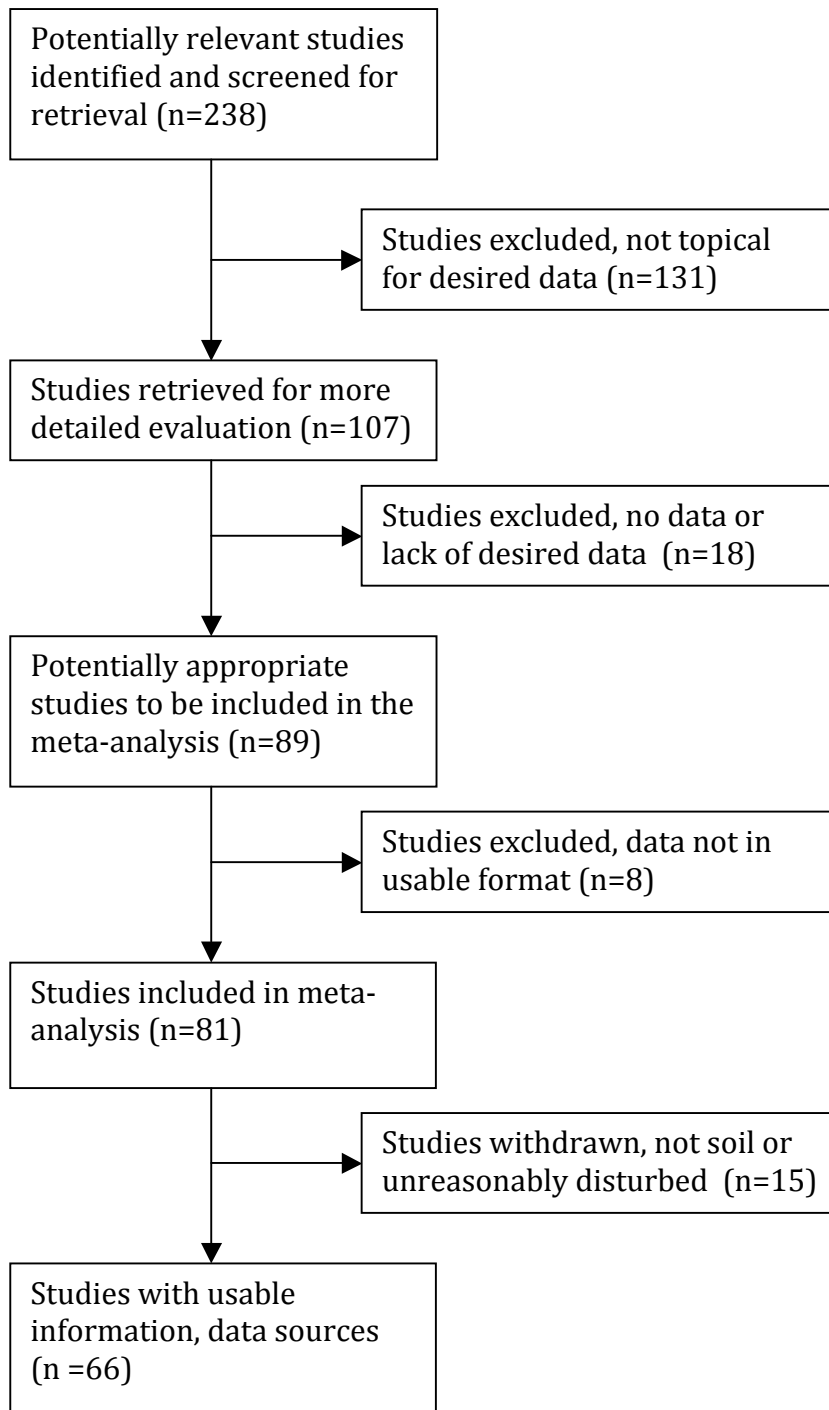

Supplement: Figure S5 — Flow of included studies for used as data sources for the meta-analysis. (PDF) [file pone.0057127.s005.pdf]
