# Supplementary material for: Differential Nutrient Limitation of Soil Microbial Biomass and Metabolic Quotients (qCO2): Is There a Biological Stoichiometry of Soil Microbes?
Source: PLoS One. 2013 Mar 19;8(3):e57127. doi: 10.1371/journal.pone.0057127 (PMC3602520; doi:10.1371/journal.pone.0057127)
Supplement: Table S1 — Summary of SMA regressions of log10-transformed C, N, and P contents in soil and microbial pools, along with predictors of soil C mineralization (CO2) and microbial metabolism ( q CO2), considering only data from forest and pasture soils. Bivariate relationships were significant (P<0.001) for all relationships shown. Slopes significantly different from one (P>0.05) are shown in boldface font. Slopes not different from one (not bold) indicate an isometric (linear) relationship among parameters. The geometric mean and standard errors (SE) of stoichiometric ratios (x∶y ratio, x∶y mean) are given for reference, but are not representative where allometric slopes different from 1. The coefficient of variation (CV) of stoichiometric ratios is given as a dimensionless index of dispersion about the mean. Single asterisks (*) indicate where different slopes are observed by considering only forest and pasture soils compared to the full range of sites (presented in Table 1), and ** indicates a different relationship was tested for only litter and organic soils (wetland organic, boreal forest, and humic horizons). (DOCX) [file pone.0057127.s006.docx]

**Table S1.** Summary of SMA regressions of log_10_-transformed C, N, and P contents in soil and microbial pools, along with predictors of soil C mineralization (CO_2_) and microbial metabolism (*q*CO_2_), considering only data from forest and pasture soils. ­­­­­

| **Analysis** | ***x*** | ***y*** | ***n*** | **r^2^** | **Int.** | **Slope** | **x:y ratio** | **x:y mean** | | | **CV** |
| --- | --- | --- | --- | --- | --- | --- | --- | --- | --- | --- | --- |
| Soil allometry | C | N | 171 | 0.80 | -1.00 | 0.94 | C:N | 15.7 | + | 0.4 | 0.3 |
|  | **C | N | 40 | 0.12 | 16.06 | **-2.85** | C:N | 36.7 | + | 4.3 | 0.7 |
|  | *C | P | 156 | 0.33 | -2.06 | 0.94 | C:P | 236.1 | + | 15 | 0.8 |
|  | *N | P | 158 | 0.49 | -1.08 | 0.99 | N:P | 14.5 | + | 0.6 | 0.5 |
| Microbial allometry | MBC | MBN | 143 | 0.71 | -1.18 | **1.13** | mC:N | 10.1 | + | 0.6 | 0.7 |
|  | *MBC | MBP | 185 | 0.48 | -2.45 | **1.43** | mC:P | 67.9 | + | 3.9 | 0.8 |
|  | MBN | MBP | 152 | 0.52 | -0.80 | 1.09 | mN:P | 6.6 | + | 0.4 | 0.8 |
| Microbial biomass | C | MBC | 177 | 0.43 | -1.45 | 0.91 | MBC:C | 2.3% | + | 0.1 | 0.6 |
|  | N | MBC | 168 | 0.53 | -0.56 | 1.00 |  |  |  |  |  |
|  | *P | MBC | 155 | 0.35 | 0.68 | 0.95 |  |  |  |  |  |
| Respiration and metabolism | MBC | CO_2_ | 63 | 0.56 | 1.62 | -4.36 |  |  |  |  |  |
|  | mC:P | *q*CO_2_ | 63 | 0.15 | -1.17 | 1.93 |  |  |  |  |  |
|  | P_i_ | *q*CO_2_ | 27 | 0.47 | 0.75 | 0.60 |  |  |  |  |  |

Bivariate relationships were significant (P < 0.001) for all relationships shown. Slopes significantly different from one (P > 0.05) are shown in boldface font. Slopes not different from one (not bold) indicate an isometric (linear) relationship among parameters. The geometric mean and standard errors (SE) of stoichiometric ratios (x:y ratio, x:y mean) are given for reference, but are not representative where allometric slopes different from 1. The coefficient of variation (CV) of stoichiometric ratios is given as a dimensionless index of dispersion about the mean. Single asterisks (*) indicate where different slopes are observed by considering only forest and pasture soils compared to the full range of sites (presented in Table 1), and ** indicates a different elationship was tested for only litter and organic soils (wetland organic, boreal forest, and humic horizons).
