# Supplementary material for: Differential Nutrient Limitation of Soil Microbial Biomass and Metabolic Quotients (qCO2): Is There a Biological Stoichiometry of Soil Microbes?
Source: PLoS One. 2013 Mar 19;8(3):e57127. doi: 10.1371/journal.pone.0057127 (PMC3602520; doi:10.1371/journal.pone.0057127)
Supplement: Table S2 — Summary of SMA regressions of log10-transformed relationships among soil C and soil stoichiometry, paired analysis of soil stoichiometric ratios, and comparisons of soil and microbial stoichiometry. SMA regression fits for these relationships correspond with data presented in Figs. S1, S2, S3. Bivariate relationships were significant (P<0.001) for all relationships shown., unless otherwise noted (n.s.). Slopes significantly different from one (P>0.05) are shown in boldface font. Slopes not different from one (not bold) indicate an isometric (linear) relationship among parameters. Analysis of relationships between soil C∶N ratios and soil C accumulation were divided by habitat given different relationships observed among different vegetation types (Fig. S1). These groupings corresponded with soil C content greater than 24% C (20,000 mmol/kg) for habitats including boreal forests, wetland organic soils, and litter, and soil C content less than 24% (all other habitats). (DOCX) [file pone.0057127.s007.docx]

**Table S2.** Summary of SMA regressions of log_10_-transformed relationships among soil C and soil stoichiometry, paired analysis of soil stoichiometric ratios, and comparisons of soil and microbial stoichiometry.

| **Analysis** | **Land Use / Vegetation** | ***x*** | ***y*** | ***n*** | **r^2^** | **Intercept** | **Slope** | **r^2^** |
| --- | --- | --- | --- | --- | --- | --- | --- | --- |
| Soil C and stoichiometry | % C < 20 | C | CN | 221 | 0.12 | 0.04 | **0.33** | 0.12 |
|  | %C > 20 | C | CN | 40 | 0.38 | -13.92 | 3.38 | 0.38 |
|  | All | C | CP | 261 | 0.72 | -0.94 | **0.91** | 0.72 |
|  | All | C | NP | 261 | 0.56 | -1.55 | **0.75** | 0.56 |
| Soil stoichiometry | All but litter | C:N | N:P | 243 | n.s. | - | - | n.s. |
|  | Litter only | C:N | N:P | 18 | 0.32 | 2.47 | **-0.58** | 0.32 |
|  | All but litter | C:P | N:P | 243 | 0.88 | -0.83 | **0.85** | 0.88 |
|  | Litter only | C:P | N:P | 18 | n.s. | - | - | n.s. |
| Soil and microbe stoichiometry | All | C:N | mC:N | 205 | n.s. | - | - | n.s. |
|  | All | C:P | mC:P | 245 | n.s. | - | - | n.s. |
|  | All | C:P | mC:P | 196 | n.s. | - | - | n.s. |

SMA regression fits for these relationships correspond with data presented in Figs. S1-S3. Bivariate relationships were significant (P < 0.001) for all relationships shown., unless otherwise noted (n.s.). Slopes significantly different from one (P > 0.05) are shown in boldface font. Slopes not different from one (not bold) indicate an isometric (linear) relationship among parameters. Analysis of relationships between soil C:N ratios and soil C accumulation were divided by habitat given different relationships observed among different vegetation types (Fig. S1). These groupings corresponded with soil C content greater than 24% C (20,000 mmol/kg) for habitats including boreal forests, wetland organic soils, and litter, and soil C content less than 24% (all other habitats).
