# Supplementary material for: Differential Nutrient Limitation of Soil Microbial Biomass and Metabolic Quotients (qCO2): Is There a Biological Stoichiometry of Soil Microbes?
Source: PLoS One. 2013 Mar 19;8(3):e57127. doi: 10.1371/journal.pone.0057127 (PMC3602520; doi:10.1371/journal.pone.0057127)
Supplement: Table S3 — Results of all pairwise SMA regressions among log10-transformed study variables including soil and microbial C, N, and P pools, and soil stoichiometric ratios. Microbial biomass C, N, and P are abbreviated MBC, MBN, and MBP. Pi is the concentration of extractable inorganic (Olsen) P (available P), and Pi∶P is the ratio of inorganic P to soil total P. Relationships among variables were compared using Standardized Major Axis (Type II) regression (SMA), except for relationships with habitat categories, which were assessed using generalized linear models (GLM). Only relationships with P>0.05 and r2 (SMA) or R2 (GLM)>0.25 are shown for clarity, except where data are displayed graphically in separate figures (boldface). Italics indicate relationships that are autocorrelated by their definition. (DOCX) [file pone.0057127.s008.docx]

**Table S3.** Results of all pairwise SMA regressions among log_10_-transformed study variables including soil and microbial C, N, and P pools, and soil stoichiometric ratios.

|  | **Lat.** | **pH** | **C** | **N** | **P** | **MBC** | **MBN** | **MBP** | **P_i_** | **C:N** | **C:P** | **N:P** | **P_i_:P** |
| --- | --- | --- | --- | --- | --- | --- | --- | --- | --- | --- | --- | --- | --- |
| **Climate** | 0.84 | - | 0.48 | 0.39 | 0.26 | 0.35 | 0.44 | 0.42 | - | - | - | - | - |
| **Veg.** | 0.41 | - | 0.60 | 0.54 | - | 0.56 | 0.60 | 0.54 | - | 0.42 | 0.63 | 0.56 | 0.36 |
| **Latitude** |  | - | 0.33 | - | - | - | 0.26 | 0.34 | - | - | - | - | - |
| **pH** |  |  | - | - | - | - | - | - | - | - | - | - | - |
| **C** |  |  |  | **0.86** | **0.18** | **0.75** | 0.70 | 0.56 | - | ***0.38*** | ***0.73*** | ***0.56*** | - |
| **N** |  |  |  |  | **0.24** | **0.73** | 0.66 | 0.51 | - | - | 0.47 | *0.55* | - |
| **P** |  |  |  |  |  | **0.32** | - | - | - | - | - | - | - |
| **MBC** |  |  |  |  |  |  | **0.84** | **0.70** | - | - | 0.49 | 0.45 | - |
| **MBN** |  |  |  |  |  |  |  | **0.71** | - | - | 0.45 | 0.42 | - |
| **MBP** |  |  |  |  |  |  |  |  | - | - | 0.42 | 0.39 | - |
| **P_i_** |  |  |  |  |  |  |  |  |  | - | - | - | *0.61* |
| **C:N** |  |  |  |  |  |  |  |  |  |  | *0.37* | - | - |
| **C:P** |  |  |  |  |  |  |  |  |  |  |  | *0.84* | - |
| **N:P** |  |  |  |  |  |  |  |  |  |  |  |  | - |
| **P_i_:P** |  |  |  |  |  |  |  |  |  |  |  |  |  |
| **mC:N** |  |  |  |  |  |  |  |  |  |  |  |  |  |
| **mC:P** |  |  |  |  |  |  |  |  |  |  |  |  |  |
| **mN:P** |  |  |  |  |  |  |  |  |  |  |  |  |  |
| **P_m_:P** |  |  |  |  |  |  |  |  |  |  |  |  |  |
| **P_m_:P_i_** |  |  |  |  |  |  |  |  |  |  |  |  |  |
| **CO_2_** |  |  |  |  |  |  |  |  |  |  |  |  |  |

Microbial biomass C, N, and P are abbreviated MBC, MBN, and MBP. P_i_ is the concentration of extractable inorganic (Olsen) P (available P), and P_i_:P is the ratio of inorganic P to soil total P. Relationships among variables were compared using Standardized Major Axis (Type II) regression (SMA), except for relationships with habitat categories, which were assessed using generalized linear models (GLM). Only relationships with P > 0.05 and r^2^ (SMA) or R^2^ (GLM) > 0.25 are shown for clarity, except where data are displayed graphically in separate figures (boldface). Italics indicate relationships that are autocorrelated by their definition.
