# Supplementary material for: Differential Nutrient Limitation of Soil Microbial Biomass and Metabolic Quotients (qCO2): Is There a Biological Stoichiometry of Soil Microbes?
Source: PLoS One. 2013 Mar 19;8(3):e57127. doi: 10.1371/journal.pone.0057127 (PMC3602520; doi:10.1371/journal.pone.0057127)
Supplement: Table S4 — Results of all pairwise SMA regressions among log10-transformed study variables including soil microbial stoichiometry, respiration, and metabolism ( q CO2). Microbial biomass C∶N, C∶P, and N∶P ratios are abbreviated by mC∶N, mC∶P, and mN∶P, respectively. The ratio of microbial P to soil total P is abbreviated Pm∶P, and Pm∶Pi is the ratio of microbial P to inorganic P. Relationships among variables were compared using Standardized Major Axis (Type II) regression (SMA), except for relationships with habitat categories, which were assessed using generalized linear models (GLM). Only relationships with P>0.05 and r2 (SMA) or R2 (GLM)>0.25 are shown for clarity, except where data are displayed graphically in separate figures (boldface). Italics indicate relationships that are autocorrelated by their definition. (DOCX) [file pone.0057127.s009.docx]

**Table S4.** Results of all pairwise SMA regressions among log_10_-transformed study variables including soil microbial stoichiometry, respiration, and metabolism (*q*CO_2_).

|  | **mC:N** | **mC:P** | **mN:P** | **P_m_:P** | **P_m_:P_i_** | **CO_2_** | ***q*CO_2_** |
| --- | --- | --- | --- | --- | --- | --- | --- |
| **Climate** | - | - | - | - | 0.44 | - | - |
| **Veg.** | - | **0.18** | - | 0.36 | 0.61 | - | 0.37 |
| **Latitude** | - | - | - | - | 0.27 | - | - |
| **pH** | - | - | - | - | - | **-** | **-** |
| **C** | - | - | - | - | 0.34 | - | - |
| **N** | - | - | - | - | 0.25 | - | - |
| **P** | - | - | - | - | - | - | - |
| **MBC** | - | - | - | 0.34 | 0.34 | **0.70** | - |
| **MBN** | - | - | - | 0.34 | 0.39 | - | - |
| **MBP** | - | - | - | *0.68* | *0.48* | 0.37 | - |
| **P_i_** | - | - | - | - | *0.26* | - | **0.44** |
| **C:N** | **-** | **-** | **-** | - | - | - | - |
| **C:P** | **-** | **-** | **-** | *0.53* | 0.42 | - | - |
| **N:P** | **-** | **-** | **-** | *0.53* | 0.38 | - | - |
| **P_i_:P** | - | - | - | - | - | - | 0.32 |
| **mC:N** |  | - | - | - | - | - | - |
| **mC:P** |  |  | *0.57* | *0.34* | - | - | **0.21** |
| **mN:P** |  |  |  | - | - | - | - |
| **P_m_:P** |  |  |  |  | *0.50* | 0.30 | - |
| **P_m_:P_i_** |  |  |  |  |  | - | - |
| **CO_2_** |  |  |  |  |  |  | *0.57* |

Microbial biomass C:N, C:P, and N:P ratios are abbreviated by mC:N, mC:P, and mN:P, respectively. The ratio of microbial P to soil total P is abbreviated P_m_:P, and P_m_:P_i_ is the ratio of microbial P to inorganic P. Relationships among variables were compared using Standardized Major Axis (Type II) regression (SMA), except for relationships with habitat categories, which were assessed using generalized linear models (GLM). Only relationships with P > 0.05 and r^2^ (SMA) or R^2^ (GLM) > 0.25 are shown for clarity, except where data are displayed graphically in separate figures (boldface). Italics indicate relationships that are autocorrelated by their definition.
