# Supplementary material for: Differential Nutrient Limitation of Soil Microbial Biomass and Metabolic Quotients (qCO2): Is There a Biological Stoichiometry of Soil Microbes?
Source: PLoS One. 2013 Mar 19;8(3):e57127. doi: 10.1371/journal.pone.0057127 (PMC3602520; doi:10.1371/journal.pone.0057127)
Supplement: Table S6 — SMA parameter estimates for simultaneous fitting of microbial biomass C and P scaling relationships by land use and vegetation categories. Regression lines are compared by category in Fig. 3B, and data and regression fits are plotted by category in Fig. S4B. The simultaneous SMA relationships for microbial biomass C and P scaling were tested for differences in intercepts (P<0.001) and slopes (P = 0.284), and significantly different intercept and slope groups were determined by multiple comparisons in SMATR v.3.0, controlling the overall error rate at p<0.05. Slopes of individual relationships significantly different from one are shown in boldface. For each category, geometric mean of C∶P ratios are presented (± SE) with their coefficient of variation (CV), and grouping by multiple comparisons using Tukey's test (p<0.05) on log10-transformed data. (DOCX) [file pone.0057127.s011.docx]

**Table S6.** SMA parameter estimates for simultaneous fitting of microbial biomass C and P scaling relationships by land use and vegetation categories.

| **Land Use / Vegetation** | ***n*** | ***r^2^*** | **Int.** | **Slope** | **Int. group** | **Slope group** | **C:P Mean** | | | **CV** | **Mean group** |
| --- | --- | --- | --- | --- | --- | --- | --- | --- | --- | --- | --- |
| Tropical For. | 16 | - | - | - | ab | a | 91 | + | 23 | 1.0 | ab |
| Coniferous For. | 34 | 0.52 | -1.96 | 1.03 | **A** | a | 101 | + | 12 | 1.5 | **A** |
| Deciduous For. | 74 | 0.41 | -2.52 | **1.44** | ab | a | 66 | + | 6 | 1.4 | ab |
| Desert | 0 | na | - | - | ab | a |  | - |  | - | - |
| Crop | 40 | 0.50 | -2.19 | **1.38** | **B** | a | 61 | + | 4 | 2.3 | ab |
| Pasture | 79 | 0.63 | -2.24 | **1.32** | **B** | a | 56 | + | 5 | 1.3 | **B** |
| Tundra | 12 | 0.40 | -2.40 | 1.26 | ab | a | 141 | + | 62 | 0.6 | ab |
| Boreal For. | 5 | ns | - | - | ab | a | 39 | + | 12 | 1.5 | ab |
| Wetland Min. | 14 | ns | - | - | ab | a | 237 | + | 147 | 0.3 | **A** |
| Wetland Org. | 13 | ns | - | - | ab | a | 105 | + | 25 | 1.2 | ab |
| For. Humus | 8 | ns | - | - | ab | a | 78 | + | 20 | 1.4 | ab |
| For. Litter | 4 | ns | - | - | ab | a | 90 | + | 30 | 1.5 | ab |

Regression lines are compared by category in Fig. 3B, and data and regression fits are plotted by category in Fig. S4B. The simultaneous SMA relationships for microbial biomass C and P scaling were tested for differences in intercepts (P < 0.001) and slopes (P = 0.284), and significantly different intercept and slope groups were determined by multiple comparisons in SMATR v.3.0, controlling the overall error rate at p < 0.05. Slopes of individual relationships significantly different from one are shown in boldface. For each category, geometric mean of C:P ratios are presented (± SE) with their coefficient of variation (CV), and grouping by multiple comparisons using Tukey’s test (p < 0.05) on log_10_-transformed data.
