# Supplementary material for: Differential Nutrient Limitation of Soil Microbial Biomass and Metabolic Quotients (qCO2): Is There a Biological Stoichiometry of Soil Microbes?
Source: PLoS One. 2013 Mar 19;8(3):e57127. doi: 10.1371/journal.pone.0057127 (PMC3602520; doi:10.1371/journal.pone.0057127)
Supplement: Table S8 — SMA parameter estimates for simultaneous fitting of microbial biomass C and N scaling relationships by climate categories. The simultaneous SMA relationships were tested for differences in intercepts (P<0.001) and slopes (P<0.001), and significantly different intercept and slope groups were determined by multiple comparisons in SMATR v.3.0, by controlling the overall error rate at p<0.05. Bivariate relationships of log10-transformed data were significant (P<0.001) for all relationships shown, unless otherwise noted due to insufficent data. Slopes significantly different from one (P>0.05) are shown in boldface font. For each category, geometric mean of N∶P ratios are presented (± SE) with their coefficient of variation (CV), and with grouping by multiple comparisons using Tukey's test (p<0.05) on log10-transformed data. (DOCX) [file pone.0057127.s013.docx]

**Table S8.** SMA parameter estimates for simultaneous fitting of microbial biomass C and N scaling relationships by climate categories.

| **Climate** | ***n*** | **r^2^** | **Int.** | **Slope** | **Int. group** | **Slope group** | **x:y Mean** | | | **CV** | **Mean group** |
| --- | --- | --- | --- | --- | --- | --- | --- | --- | --- | --- | --- |
| Tropical | 14 | 0.86 | -1.51 | **1.36** | - | - | 7.9 | + | 0.6 | 0.3 | ab |
| Subtropical | 57 | 0.74 | -1.17 | 1.09 | - | - | 13.7 | + | 2.0 | 1.1 | **A** |
| Savanna | 30 | 0.62 | -1.58 | **1.41** | - | - | 14.1 | + | 2.1 | 0.8 | **A** |
| Desert | 0 | - | - | - | - | - |  | - |  | - | - |
| Temperate | 127 | 0.84 | -0.96 | 1.02 | - | - | 8.9 | + | 0.3 | 0.4 | **B** |
| Boreal | 8 | 0.70 | -0.35 | 0.74 | - | - | 11.6 | + | 1.1 | 0.3 | ab |
| Tundra | 1 | - | - | - | - | - | 13.0 |  |  | - | - |

The simultaneous SMA relationships were tested for differences in intercepts (P < 0.001) and slopes (P < 0.001), and significantly different intercept and slope groups were determined by multiple comparisons in SMATR v.3.0, by controlling the overall error rate at p < 0.05. Bivariate relationships of log_10_-transformed data were significant (P < 0.001) for all relationships shown, unless otherwise noted due to insufficent data . Slopes significantly different from one (P > 0.05) are shown in boldface font. For each category, geometric mean of N:P ratios are presented (± SE) with their coefficient of variation (CV), and with grouping by multiple comparisons using Tukey’s test (p < 0.05) on log_10_-transformed data.
