# Supplementary material for: Differential Nutrient Limitation of Soil Microbial Biomass and Metabolic Quotients (qCO2): Is There a Biological Stoichiometry of Soil Microbes?
Source: PLoS One. 2013 Mar 19;8(3):e57127. doi: 10.1371/journal.pone.0057127 (PMC3602520; doi:10.1371/journal.pone.0057127)
Supplement: Table S9 — SMA parameter estimates for simultaneous fitting of microbial biomass C and P scaling relationships by climate categories. The simultaneous SMA relationships were tested for differences in intercepts (P<0.001) and slopes (P<0.001), and significantly different intercept and slope groups were determined by multiple comparisons in SMATR v.3.0, by controlling the overall error rate at p<0.05. Bivariate relationships of log10-transformed data were significant (P<0.001) for all relationships shown, unless otherwise noted due to insufficent data. Slopes significantly different from one (P>0.05) are shown in boldface font. For each category, geometric mean of N∶P ratios are presented (± SE) with their coefficient of variation (CV), and with grouping by multiple comparisons using Tukey's test (p<0.05) on log10-transformed data. (DOCX) [file pone.0057127.s014.docx]

**Table S9.** SMA parameter estimates for simultaneous fitting of microbial biomass C and P scaling relationships by climate categories.

| **Climate** | ***n*** | **r^2^** | **Int.** | **Slope** | **Int. group** | **Slope group** | **x:y Mean** | | | **CV** | **Mean group** |
| --- | --- | --- | --- | --- | --- | --- | --- | --- | --- | --- | --- |
| Tropical | 16 | 0.01 | 0.95 | -0.62 | - | **A** | 90.1 | + | 23.5 | 1.0 | ab |
| Subtropical | 65 | 0.53 | -1.71 | 0.89 | - | **A** | 146.9 | + | 59.1 | 3.2 | **A** |
| Savanna | 33 | 0.67 | -2.39 | **1.49** | - | **B** | 66.3 | + | 5.1 | 0.4 | ab |
| Desert | 0 | - | - | - | - | - |  | - |  | - | - |
| Temperate | 176 | 0.55 | -2.11 | 1.20 | - | ab | 66.0 | + | 4.0 | 0.8 | **B** |
| Boreal | 8 | 0.15 | -4.12 | 1.89 | - | ab | 77.2 | + | 25.4 | 0.9 | ab |
| Tundra | 1 | - | - | - | - | - | 61.6 |  |  | - | - |

The simultaneous SMA relationships were tested for differences in intercepts (P < 0.001) and slopes (P < 0.001), and significantly different intercept and slope groups were determined by multiple comparisons in SMATR v.3.0, by controlling the overall error rate at p < 0.05. Bivariate relationships of log_10_-transformed data were significant (P < 0.001) for all relationships shown, unless otherwise noted due to insufficent data. Slopes significantly different from one (P > 0.05) are shown in boldface font. For each category, geometric mean of N:P ratios are presented (± SE) with their coefficient of variation (CV), and with grouping by multiple comparisons using Tukey’s test (p < 0.05) on log_10_-transformed data.
