# Supplementary material for: Differential Nutrient Limitation of Soil Microbial Biomass and Metabolic Quotients (qCO2): Is There a Biological Stoichiometry of Soil Microbes?
Source: PLoS One. 2013 Mar 19;8(3):e57127. doi: 10.1371/journal.pone.0057127 (PMC3602520; doi:10.1371/journal.pone.0057127)
Supplement: Table S10 — SMA parameter estimates for simultaneous fitting of microbial biomass N and P scaling relationships by climate categories. The simultaneous SMA relationships were tested for differences in intercepts (P<0.001) and slopes (P<0.001), and significantly different intercept and slope groups were determined by multiple comparisons in SMATR v.3.0, by controlling the overall error rate at p<0.05. Bivariate relationships of log10-transformed data were significant (P<0.001) for all relationships shown. Slopes significantly different from one (P>0.05) are shown in boldface font. For each category, geometric mean of N∶P ratios are presented (± SE) with their coefficient of variation (CV), and with grouping by multiple comparisons using Tukey's test (p<0.05) on log10-transformed data. (DOCX) [file pone.0057127.s015.docx]

**Table S10.** SMA parameter estimates for simultaneous fitting of microbial biomass N and P scaling relationships by climate categories.

| **Climate** | ***n*** | **r^2^** | **Int.** | **Slope** | **Int. group** | **Slope group** | **x:y Mean** | | | **CV** | **Mean group** |
| --- | --- | --- | --- | --- | --- | --- | --- | --- | --- | --- | --- |
| Tropical | 18 | 0.54 | -1.21 | 1.19 | **A** | ab | 16.9 | + | 3.9 | 1.0 | **A** |
| Subtropical | 57 | 0.45 | -0.75 | **0.81** | ab | **A** | 15.2 | + | 5.5 | 2.7 | ab |
| Savanna | 30 | 0.76 | -0.75 | 1.16 | **B** | ab | 5.9 | + | 0.5 | 0.5 | **B** |
| Boreal | 14 | 0.50 | -2.75 | **2.29** | ab | **B** | 5.2 | + | 1.0 | 0.7 | **B** |
| Temperate | 136 | 0.44 | -0.72 | 1.00 | **B** | ab | 6.6 | + | 0.4 | 0.7 | **B** |
| Desert | 6 | 0.75 | -0.65 | **0.59** | ab | ab | 7.2 | + | 0.8 | 0.3 | ab |
| Tundra | 8 | 0.94 | -0.13 | **0.69** | **C** | **A** | 3.9 | + | 0.2 | 0.1 | **B** |
|  |  |  |  |  |  |  |  |  |  |  |  |

The simultaneous SMA relationships were tested for differences in intercepts (P < 0.001) and slopes (P < 0.001), and significantly different intercept and slope groups were determined by multiple comparisons in SMATR v.3.0, by controlling the overall error rate at p < 0.05. Bivariate relationships of log_10_-transformed data were significant (P < 0.001) for all relationships shown. Slopes significantly different from one (P > 0.05) are shown in boldface font. For each category, geometric mean of N:P ratios are presented (± SE) with their coefficient of variation (CV), and with grouping by multiple comparisons using Tukey’s test (p < 0.05) on log_10_-transformed data.
