# Supplementary material for: Differential Nutrient Limitation of Soil Microbial Biomass and Metabolic Quotients (qCO2): Is There a Biological Stoichiometry of Soil Microbes?
Source: PLoS One. 2013 Mar 19;8(3):e57127. doi: 10.1371/journal.pone.0057127 (PMC3602520; doi:10.1371/journal.pone.0057127)
Supplement: Table S11 — SMA parameter estimates for simultaneous fitting of homeostatic relationships between microbial and soil stoichiometry by vegetation categories. The simultaneous SMA relationships were tested for differences in intercepts (P<0.001) and slopes (P<0.001). Slopes significantly different from one (P>0.05) are shown in boldface font. Only significant relationships with r2≥0.3 and n>5 are shown. (DOCX) [file pone.0057127.s016.docx]

**Table S11.** SMA parameter estimates for simultaneous fitting of homeostatic relationships between microbial and soil stoichiometry by vegetation categories.

| **y** | **x** | **Land Use / Vegetation** | ***n*** | **r^2^** | **Int.** | **Slope** |
| --- | --- | --- | --- | --- | --- | --- |
| mC:N | C:N | Tropical For. | 13 | 0.44 | 1.37 | **-0.47** |
|  |  | Coniferous For. | 9 | 0.01 | - | **-** |
|  |  | Deciduous For. | 64 | 0.03 | - | **-** |
|  |  | Desert | 0 | - | - | **-** |
|  |  | Crop | 24 | 0.05 | - | **-** |
|  |  | Pasture | 54 | 0.02 | - | **-** |
|  |  | Tundra | 12 | 0.19 | - | **-** |
|  |  | Boreal For. | 5 | 0.09 | - | **-** |
|  |  | Wetland Min. | 10 | 0.81 | 3.13 | **-1.99** |
|  |  | Wetland Org. | 7 | 0.03 | - | **-** |
|  |  | For. Humus | 5 | 0.01 | - | **-** |
|  |  | For. Litter | 2 | - | - | **-** |
| mC:P | C:P | Tropical For. | 15 | 0.18 | - | **-** |
|  |  | Coniferous For. | 27 | 0.06 | - | **-** |
|  |  | Deciduous For. | 60 | 0.25 | - | **-** |
|  |  | Desert | 0 | - | - | **-** |
|  |  | Crop | 26 | 0.07 | - | **-** |
|  |  | Pasture | 70 | 0.01 | - | **-** |
|  |  | Tundra | 11 | 0.68 | -3.27 | **1.94** |
|  |  | Boreal For. | 3 | 0.83 | - | **-** |
|  |  | Wetland Min. | 11 | 0.06 | - | **-** |
|  |  | Wetland Org. | 12 | 0.01 | - | **-** |
|  |  | For. Humus | 8 | 0.52 | -2.73 | 1.42 |
|  |  | For. Litter | 4 | 0.72 | - | **-** |
| mN:P | N:P | Tropical For. | 14 | 0.13 | - | **-** |
|  |  | Coniferous For. | 10 | 0.19 | - | **-** |
|  |  | Deciduous For. | 58 | 0.03 | - | **-** |
|  |  | Desert | 0 | - | - | **-** |
|  |  | Crop | 22 | 0.25 | 0.91 | **-0.42** |
|  |  | Pasture | 49 | 0.17 | - | **-** |
|  |  | Tundra | 11 | 0.85 | -2.10 | **2.14** |
|  |  | Boreal For. | 3 | 0.05 | - | **-** |
|  |  | Wetland Min. | 10 | 0.28 | - | **-** |
|  |  | Wetland Org. | 6 | 0.27 | - | **-** |
|  |  | For. Humus | 8 | 0.00 | - | **-** |
|  |  | For. Litter | 5 | 0.00 | - | **-** |

The simultaneous SMA relationships were tested for differences in intercepts (P < 0.001) and slopes (P < 0.001). Slopes significantly different from one (P > 0.05) are shown in boldface font. Only significant relationships with r^2^ > 0.3 and n > 5 are shown.
