# Supplementary material for: Differential Nutrient Limitation of Soil Microbial Biomass and Metabolic Quotients (qCO2): Is There a Biological Stoichiometry of Soil Microbes?
Source: PLoS One. 2013 Mar 19;8(3):e57127. doi: 10.1371/journal.pone.0057127 (PMC3602520; doi:10.1371/journal.pone.0057127)
Supplement: Table S12 — SMA parameter estimates for simultaneous fitting of homeostatic relationships between microbial and soil stoichiometry by climate categories. Microbial biomass C∶N, C∶P, and N∶P ratios are abbreviated by mC∶N, mC∶P, and mN∶P, respectively. The simultaneous SMA relationships were tested for differences in intercepts (P<0.001) and slopes (P<0.001). Slopes significantly different from one (P>0.05) are shown in boldface font. Only significant relationships with r2≥0.3 and n>5 are shown. (DOCX) [file pone.0057127.s017.docx]

**Table S12.** SMA parameter estimates for simultaneous fitting of homeostatic relationships between microbial and soil stoichiometry by climate categories.

| **y** | **x** | **Climate** | ***n*** | **r^2^** | **Int.** | **Slope** |
| --- | --- | --- | --- | --- | --- | --- |
| mC:N | C:N | Tropical | 13 | 0.41 | -0.44 | 1.08 |
|  |  | Subtropical | 41 | 0.03 | - | - |
|  |  | Savanna | 30 | 0.14 | - | - |
|  |  | Desert | 0 | - | - | - |
|  |  | Temperate | 112 | 0.05 | - | - |
|  |  | Boreal | 8 | 0.42 | 1.79 | **-0.52** |
|  |  | Tundra | 0 | - | - | - |
| mC:P | C:P | Tropical | 15 | 0.21 | - | - |
|  |  | Subtropical | 40 | 0.10 | - | - |
|  |  | Savanna | 32 | 0.14 | - | - |
|  |  | Desert | 0 | - | - | - |
|  |  | Temperate | 155 | 0.00 | - | - |
|  |  | Boreal | 5 | 0.89 | 12.2 | **-3.40** |
|  |  | Tundra | 0 | - | - | - |
| mN:P | N:P | Tropical | 14 | 0.08 | - | - |
|  |  | Subtropical | 32 | 0.02 | - | - |
|  |  | Savanna | 30 | 0.12 | - | - |
|  |  | Desert | 0 | - | - | - |
|  |  | Temperate | 115 | 0.01 | - | - |
|  |  | Boreal | 5 | 0.92 | -3.17 | **2.37** |
|  |  | Tundra | 0 | - | - | - |

Microbial biomass C:N, C:P, and N:P ratios are abbreviated by mC:N, mC:P, and mN:P, respectively. The simultaneous SMA relationships were tested for differences in intercepts (P < 0.001) and slopes (P < 0.001). Slopes significantly different from one (P > 0.05) are shown in boldface font. Only significant relationships with r^2^ > 0.3 and n > 5 are shown.
