# Supplementary material for: Differential Nutrient Limitation of Soil Microbial Biomass and Metabolic Quotients (qCO2): Is There a Biological Stoichiometry of Soil Microbes?
Source: PLoS One. 2013 Mar 19;8(3):e57127. doi: 10.1371/journal.pone.0057127 (PMC3602520; doi:10.1371/journal.pone.0057127)
Supplement: Table S13 — Multivariate general linear regression models of soil C mineralization rates (CO2) as a function of ecosystem, soil and microbial factors. General linear models were compared using an exhaustive search, but only selected models with all predictors simultaneously significant are shown. To account for differences in missing data among parameters, we computed the test statistic %Var = R2 * (n samples in model/n total samples). Model 9 (bold) had the greatest %Var, explaining more variance in more data points. Notably, addition of soil pH to models (Models 10–14) improved fit (R2), but at the cost of fewer observations (df), resulting in a lower % Var. Models including both pH and inorganic P (Pi) had far fewer observations (Models 15–19), and consequently lower % Var explained. Linear regression results for individual predictors of CO2 by Standardized Major Axis regression (SMA) are given in Table S4. (DOCX) [file pone.0057127.s018.docx]

**Table S13.** Multivariate general linear regression models of soil C mineralization rates (CO_2_) as a function of ecosystem, soil and microbial factors.

| **#** | **Model** | **R^2^** | **AIC** | **df** | **% Var** |
| --- | --- | --- | --- | --- | --- |
| 1 | CO_2_ ~ MBC | 0.697 | 91.7 | 91 | 70 |
| 2 | CO_2_ ~ MBC + pH | 0.729 | 66.8 | 70 | 56 |
| 3 | CO_2_ ~ MBC + C:P | 0.735 | 72.1 | 86 | 69 |
| 4 | CO_2_ ~ MBC + mC:P | 0.743 | 77.5 | 91 | 74 |
| 5 | CO_2_ ~ MBC + mC:P + C:P + Climate | 0.778 | 60.5 | 86 | 74 |
| 6 | CO_2_ ~ MBC + mC:P + C:P + MBC*C:P | 0.804 | 47.6 | 86 | 76 |
| 7 | CO_2_ ~ MBC + mC:P + Vegetation | 0.806 | 60.6 | 91 | 81 |
| 8 | CO_2_ ~ MBC + mC:P + C:P + Climate + MBC*C:P | 0.819 | 43.7 | 86 | 77 |
| **9** | **CO_2_** **~ MBC + mC:P + Vegetation + Climate** | **0.828** | **51.2** | **91** | **83** |
| 10 | CO_2_ ~ MBC + Vegetation + pH | 0.835 | 39.5 | 70 | 64 |
| 11 | CO_2_ ~ MBC + mC:P + C:P + MBC*C:P + pH | 0.836 | 27.9 | 65 | 60 |
| 12 | CO_2_ ~ MBC + mC:P + Vegetation + pH | 0.864 | 26.4 | 70 | 66 |
| 13 | CO_2_ ~ MBC + mC:P + Vegetation + pH + Lat | 0.879 | 20.1 | 69 | 67 |
| 14 | CO_2_ ~ MBC + mC:P + Vegetation + Climate + pH | 0.882 | 18.6 | 70 | 68 |
| 15 | CO_2_ ~ pH + N:P + P_i_ + mN:P | 0.885 | 9.5 | 13 | 13 |
| 16 | CO_2_ ~ pH + C:P + P_i_ + MBC | 0.897 | -0.5 | 23 | 23 |
| 17 | CO_2_ ~ pH + N:P + P_i_ + MBC | 0.902 | -1.6 | 23 | 23 |
| 18 | CO_2_ ~ pH + N:P + MBC + P_i_ + MBC* P_i_ | 0.935 | -10.7 | 23 | 24 |
| 19 | CO_2_ ~ pH + C:P + MBC + P_i_ + MBC* P_i_ | 0.935 | -10.8 | 23 | 24 |

General linear models were compared using an exhaustive search, but only selected models with all predictors simultaneously significant are shown. To account for differences in missing data among parameters, we computed the test statistic %Var = R^2^ * (n samples in model / n total samples). Model 9 (bold) had the greatest %Var, explaining more variance in more data points. Notably, addition of soil pH to models (Models 10-14) improved fit (R^2^), but at the cost of fewer observations (df), resulting in a lower % Var. Models including both pH and inorganic P (P_i_) had far fewer observations (Models 15-19), and consequently lower % Var explained. Linear regression results for individual predictors of CO_2_ by Standardized Major Axis regression (SMA) are given in Table S4.
