# Supplementary material for: Differential Nutrient Limitation of Soil Microbial Biomass and Metabolic Quotients (qCO2): Is There a Biological Stoichiometry of Soil Microbes?
Source: PLoS One. 2013 Mar 19;8(3):e57127. doi: 10.1371/journal.pone.0057127 (PMC3602520; doi:10.1371/journal.pone.0057127)
Supplement: Table S14 — Multivariate general linear regression models of the microbial metabolic quotient qCO2 as a function of ecosystem, soil and microbial factors. General linear models were compared using an exhaustive search, but only selected models with all predictors simultaneously significant are shown. To account for differences in missing data among parameters, we computed the test statistic %Var = R2 * (n samples in model/n total samples). Model 9 had the greatest %Var and lowest AIC. Linear regression results for individual predictors of qCO2 by Standardized Major Axis regression (SMA) are given in Table S4. (DOCX) [file pone.0057127.s019.docx]

Table S14. Multivariate general linear regression models of the microbial metabolic quotient *qCO_2_* as a function of ecosystem, soil and microbial factors.

| **#** | **Model** | **R^2^** | **AIC** | **df** | **% Var** |
| --- | --- | --- | --- | --- | --- |
| 1 | *q*CO_2_ ~ mC:P+C:P | 0.243 | 64 | 86 | 23 |
| 2 | *q*CO_2_ ~ mC:P+pH+Lat | 0.422 | 47.4 | 69 | 32 |
| 3 | *q*CO_2_ ~ pH + C + P_i_ + C:P | 0.531 | -1.1 | 23 | 13 |
| 4 | *q*CO_2_ ~ mC:P+C:P+mC:P*C:P+pH | 0.540 | 37.9 | 65 | 39 |
| 5 | *q*CO_2_ ~ pH+mC:P+Vegetation | 0.610 | 26.4 | 70 | 47 |
| 6 | *q*CO_2_ ~ pH+mC:P+Vegetation + Climate | 0.664 | 22.8 | 70 | 51 |
| 7 | *q*CO_2_ ~ pH+mC:P+N:P+Vegetation | 0.705 | 5.9 | 65 | 50 |
| 8 | *q*CO_2_ ~ mC:P+C:P+pH+Vegetation+Climate | 0.765 | -9.4 | 65 | 55 |
| **9** | ***q*CO_2_ ~ mC:P+N:P+pH+Vegetation+Climate** | **0.777** | **-11.1** | **65** | **56** |

General linear models were compared using an exhaustive search, but only selected models with all predictors simultaneously significant are shown. To account for differences in missing data among parameters, we computed the test statistic %Var = R^2^ * (n samples in model / n total samples). Model 9 had the greatest %Var and lowest AIC. Linear regression results for individual predictors of *q*CO_2_ by Standardized Major Axis regression (SMA) are given in Table S4.
