# Supplementary material for: Differential Nutrient Limitation of Soil Microbial Biomass and Metabolic Quotients (qCO2): Is There a Biological Stoichiometry of Soil Microbes?
Source: PLoS One. 2013 Mar 19;8(3):e57127. doi: 10.1371/journal.pone.0057127 (PMC3602520; doi:10.1371/journal.pone.0057127)
Supplement: Table S15 — Publications used as sources of soil and microbial element pool data. Ref. no. refers to reference citation number for this article, with most data source publication references given in Text S1. Data Ref. no. indicates the numbering used in the extracted data set presented in Table S18. (DOCX) [file pone.0057127.s020.docx]

**Table S15.** Publications used as sources of soil and microbial element pool data.

| **Climate** | **Ref. no.** | **Data Ref. no.** | **Reference** | **Land Use(s)** | **n. obvs.** |
| --- | --- | --- | --- | --- | --- |
| Tropical | [97] | 1 | Barbhuiya et al. (2008) | Tropical Forest | 1 |
|  | [52] | 2 | Cleveland et al. (2004) | Tropical Forest | 4 |
|  | [98] | 3 | Pandey and Srivastava (2009) | Tropical Forest | 2 |
|  | [99] | 4 | Yavitt et al. (1993) | Tropical Forest | 1 |
|  | [100] | 5 | Gangcai et al. (2001) | Coniferous Forest | 3 |
|  | [101] | 6 | Monkiedje et al. (2006) | Crop | 3 |
|  | [102] | 7 | Singh and Singh (1995) | Crop | 6 |
|  |  |  |  | **Total** | **20** |
|  |  |  |  |  |  |
| Subtropical | [103] | 8 | Barbhuiya et al. (2004) | Tropical Forest | 4 |
|  | [104] | 9 | Arunachalam et al. (1996) | Coniferous Forest | 1 |
|  | [105] | 10 | Chen and He (2004) | Multiple | 6 |
|  | [106] | 11 | Wang and Wang (2008) | Coniferous Forest | 3 |
|  | [107] | 12 | Wang et al. (2004) | Coniferous Forest | 2 |
|  | [108] | 13 | Arunachalam and Arunachalam (2000) | Deciduous Forest | 6 |
|  | [53] | 14 | Devi and Yadava (2006) | Deciduous Forest | 2 |
|  | [109] | 15 | Maithani et al. (1996) | Deciduous Forest | 3 |
|  | [110] | 16 | Balota et al. (2003) | Crop | 12 |
|  | [111] | 17 | Patra et al. (1995) | Crop | 2 |
|  | [112] | 18 | Prasad et al. (1995) | Crop | 3 |
|  | [113] | 19 | Sharma et al. (2004) | Crop | 5 |
|  | [114] | 20 | Srivastava (1998) | Crop | 8 |
|  | [115] | 21 | Srivastava and Singh (1988) | Crop | 4 |
|  | [85] | 22 | Li et al. (2006) | Wetland Mineral | 5 |
|  | [95] | 23 | Schilling and Lockaby (2005) | Wetland Mineral | 12 |
|  | [116] | 24 | Schilling et al. (1999) | Wetland Mineral | 2 |
|  | [117] | 25 | Tang et al. (2007) | Wetland Mineral | 2 |
|  | [118] | 26 | Corstanje et al. (2007) | Wetland Organic | 3 |
|  | [86] | 27 | McLatchey and Reddy (1998) | Wetland Organic | 1 |
|  | [119] | 28 | Reddy et al. (1998) | Wetland Organic | 4 |
|  |  |  |  | **Total** | **90** |

Ref. no. refers to reference citation number for this article, with most data source publication references given in Text S1. Data Ref. no. indicates the numbering used in the extracted data set presented in Table S18.**Table S15** (continued-2/3):

| **Climate** | **Ref. no.** | **Data Ref. no.** | **Reference** | **Land Use(s)** | **n. obvs.** |
| --- | --- | --- | --- | --- | --- |
| Savanna | [120] | 29 | Oberson et al. (2001) | Forest, Pasture, Wetland | 3 |
|  | [121] | 30 | Roy and Singh (1994) | Deciduous Forest | 2 |
|  | [122] | 31 | Singh et al. (1991) | Deciduous Forest | 4 |
|  | [123] | 32 | Khan and Joergensen (2006) | Crop | 11 |
|  | [124] | 33 | Srivastava and Lal (1994) | Crop | 1 |
|  | [125] | 34 | Agbenin and Adeniyi (2005) | Pasture | 5 |
|  | [126] | 35 | Kwabiah et al. (2003) | Pasture | 1 |
|  |  |  |  | **Total** | **27** |
|  |  |  |  |  |  |
| Desert | [127] | 36 | Lajtha and Schlesinger (1988) | Scrub | 4 |
|  | [128] | 37 | Sarig et al. (1996) | Scrub | 6 |
|  |  |  |  | **Total** | **10** |
|  |  |  |  |  |  |
| Temperate | [46] | 38 | Chen et al. (2004) | Coniferous Forest, Pasture | 30 |
|  | [129] | 39 | Christ et al. (1997) | Coniferous Forest | 1 |
|  | [130] | 40 | Lorenz et al. (2001) | Coniferous Forest | 4 |
|  | [131] | 41 | Joergensen et al. (1995) | Deciduous Forest | 38 |
|  | [132] | 42 | Wright and Coleman (2000) | Deciduous Forest | 8 |
|  | [133] | 43 | Meyer et al. (1997) | Crop | 2 |
|  | [134] | 44 | Quintern et al. (2006) | Crop | 3 |
|  | [135] | 45 | McIntosh et al. (1999) | Pasture | 1 |
|  | [136] | 46 | Ross et al. (1997) | Pasture | 4 |
|  | [137] | 47 | Saggar et al. (1999) | Pasture | 1 |
|  | [138] | 48 | Saggar et al. (2000) | Pasture | 3 |
|  | [139] | 49 | Sarathchandra et al. (1989) | Pasture | 1 |
|  | [140] | 50 | Turner et al. (2001) | Pasture | 29 |
|  | [141] | 51 | West et al. (1986) | Pasture | 2 |
|  | [142] | 52 | Holland (2006) | Heath | 9 |
|  | [143] | 53 | Kopáček et al. (2004) | Heath | 2 |
|  | [144] | 54 | Khan et al. (2007) | Wetland Mineral | 2 |
|  | [87] | 55 | Brake et al. (1999) | Wetland Organic | 6 |

**Table S15** (continued 3/3):

| **Climate** | **Ref. no.** | **Data Ref. no.** | **Reference** | **Land Use(s)** | **n. obvs.** |
| --- | --- | --- | --- | --- | --- |
| Temperate (continued) | [45] | 56 | Chen et al. (2000) | Coniferous Forest, Pasture | 6 |
|  | [55] | 57 | Chen et al. (2003) | Coniferous Forest, Pasture | 4 |
|  | [44] | 58 | Ross et al. (1999) | Forests, Pasture | 7 |
|  | [145] | 59 | Saggar et al. (1998) | Coniferous Forest, Pasture | 1 |
|  | [146] | 60 | Santrucková et al. (2004) | Coniferous Forest | 9 |
|  | [84] | 61 | Sparling et al. (1994) | Coniferous Forest | 11 |
|  |  |  |  | **Total** | **184** |
|  |  |  |  |  |  |
| Boreal | [147] | 62 | Chu and Grogan (2009) | Boreal Forest, Wetland | 4 |
|  | [148] | 63 | Lagerström et al. (2009) | Boreal Forest | 3 |
|  | [149] | 64 | Larsen et al. (2007) | Boreal Forest | 13 |
|  | [51] | 65 | Schmidt et al. (2002) | Wetland Organic | 2 |
|  | [96] | 66 | Jonasson et al. (1996) | Tundra | 2 |
|  |  |  |  | **Total** | **24** |
|  |  |  |  |  |  |
|  |  |  |  | **Global Total** | **355** |
