# Supplementary material for: Differential Nutrient Limitation of Soil Microbial Biomass and Metabolic Quotients (qCO2): Is There a Biological Stoichiometry of Soil Microbes?
Source: PLoS One. 2013 Mar 19;8(3):e57127. doi: 10.1371/journal.pone.0057127 (PMC3602520; doi:10.1371/journal.pone.0057127)
Supplement: Table S16 — Codes for climate categories used to describe soils in the full microbial stoichiometry data set (Table S18). (DOCX) [file pone.0057127.s021.docx]

**Table S16.** Codes for climate categories used to describe soils in the full microbial stoichiometry data set (Table S18).

| **Climate Class** | **Climate** |
| --- | --- |
| 1 | Tropical |
| 2 | Subtropical |
| 3 | Savanna |
| 4 | Desert |
| 5 | Temperate |
| 6 | Boreal |
| 7 | Tundra |
