# Supplementary material for: Differential Nutrient Limitation of Soil Microbial Biomass and Metabolic Quotients (qCO2): Is There a Biological Stoichiometry of Soil Microbes?
Source: PLoS One. 2013 Mar 19;8(3):e57127. doi: 10.1371/journal.pone.0057127 (PMC3602520; doi:10.1371/journal.pone.0057127)
Supplement: Table S17 — Codes used to describe land use and vegetation classification of soils in the full microbial stoichiometry data set (Table S18). (DOCX) [file pone.0057127.s022.docx]

**Table S17.** Codes used to describe land use and vegetation classification of soils in the full microbial stoichiometry data set (Table S18).

| **Land Use Class** | **Land Use** |
| --- | --- |
| 1 | Tropical Forest |
| 2 | Coniferous Forest |
| 3 | Deciduous Forest |
| 4 | Dry Scrub |
| 5 | Crop |
| 6 | Pasture |
| 7 | Tundra Heath |
| 8 | Boreal Forest |
| 9 | Wetland, Mineral Soil |
| 10 | Wetland, Organic Soil |
| 11 | Forest Floor, Coniferous |
| 12 | Forest Floor, Deciduous |
| 13 | Litter, Coniferous Forest |
| 14 | Litter, Deciduous Forest |
