# Supplementary material for: Differential Nutrient Limitation of Soil Microbial Biomass and Metabolic Quotients (qCO2): Is There a Biological Stoichiometry of Soil Microbes?
Source: PLoS One. 2013 Mar 19;8(3):e57127. doi: 10.1371/journal.pone.0057127 (PMC3602520; doi:10.1371/journal.pone.0057127)
Supplement: Text S1 — Additional references for publications used as data sources. (DOCX) [file pone.0057127.s024.docx]

**Text S1.** Additional references for publications used as data sources.

97. Barbhuiya AR, Arunachalam A, Pandey HN, Arunachalam K, Khan ML (2008) Effects of anthropogenic disturbance on soil microbial biomass С, N and P in a tropical rainforest ecosystem of Assam, Northeast India. Malaysian Journal of Soil Science 12: 31-44.

98. Pandey CB, Srivastava RC (2009) Plant available phosphorus in homegarden and native forest soils under high rainfall in an equatorial humid tropics. Plant and Soil 316: 71-80.

99. Yavitt JB, Wieder RK, Wright SJ (1993) Soil nutrient dynamics in response to irrigation of a Panamanian tropical moist forest. Biogeochemistry 19: 1-25.

100. Gangcai C, Lu G, Shilu W, Yangou W, Goujiang W (2001) A comparative study on the microbiological characteristics of soils under different land—use conditions from karst areas of southwest China. Chinese Journal of Geochemistry 20: 52–58.

101. Monkiedje A, Spiteller M, Fotio D, Sukul P (2006) The effect of land use on soil health indicators in peri-Urban agriculture in the humid forest zone of southern Cameroon. Journal of Environmental Quality 35: 2402-2409.

102. Singh S, Singh JS (1995) Microbial biomass associated with water-stable aggregates in forest, savanna and cropland soils of a seasonally dry tropical region, India. Soil Biology and Biochemistry 27: 1027–1033.

103. Barbhuiya AR, Arunachalam A, Pandey HN, Arunachalam K, Khan ML, et al. (2004) Dynamics of soil microbial biomass C, N and P in disturbed and undisturbed stands of a tropical wet-evergreen forest. European Journal of Soil Biology 40: 113–121.

104. Arunachalam A, Maithani K, Pandey HN, Tripathi RS (1996) The impact of disturbance on detrital dynamics and soil microbial biomass of a Pinus kesiya forest in north-east India. Forest Ecology and Management 88: 273–282.

105. Chen GC, He ZL (2004) Determination of soil microbial biomass phosphorus in acid red soils from southern China. Biology and Fertility of Soils 39: 446-451.

106. Wang Q-K, Wang S-L (2008) Soil microbial properties and nutrients in pure and mixed Chinese fir plantations. Journal of Forestry Research 19: 131-135.

107. Wang FE, Chen YX, Tian GM, Kumar S, He YF, et al. (2004) Microbial biomass carbon, nitrogen and phosphorus in the soil profiles of different vegetation covers established for soil rehabilitation in a red soil region of southeastern China. Nutrient Cycling in Agroecosystems 68: 181–189.

108. Arunachalam A, Arunachalam K (2000) Influence of gap size and soil properties on microbial biomass in a subtropical humid forest of north-east India. Plant and Soil 223: 187–195.

109. Maithani K, Tripathi RS, Arunachalam A, Pandey HN (1996) Seasonal dynamics of microbial biomass C, N and P during regrowth of a disturbed subtropical humid forest in north-east India. Applied Soil Ecology 4: 31–37.

110. Balota EL, Colozzi-Filho A, Andrade DS, Dick RP (2003) Microbial biomass in soils under different tillage and crop rotation systems. Biology and Fertility of Soils 38: 15-20.

111. Patra DD, Chand S, Anwar M (1995) Seasonal changes in microbial biomass in soils cropped with palmarosa (*Cymbopogon martinii* L.) and Japanese mint (*Mentha arvensis* L.) in subtropical India. Biology and Fertility of Soils 19: 193–196.

112. Prasad P, Basu S, Behera N (1995) A comparative account of the microbiological characteristics of soils under natural forest, grassland and cropfield from Eastern India. Plant and Soil 175: 85–91.

113. Sharma P, Rai SC, Sharma R, Sharma E (2004) Effects of land-use change on soil microbial C, N and P in a Himalayan watershed. Pedobiologia 48: 83–92.

114. Srivastava SC (1998) Microbial contribution to extractable N and P after air-drying of dry tropical soils. Biology and Fertility of Soils 26: 31-34.

115. Srivastava SC, Singh JS (1988) Carbon and phosphorus in the soil biomass of some tropical soils of India. Soil Biology and Biochemistry 20: 743-747.

116. Schilling EB, Lockaby BG, Rummer R (1999) Belowground nutrient dynamics following three harvest intensities on the Pearl River floodplain, Mississippi. Soil Science Society of America Journal 63: 1856-1868.

117. Tang G, Xiao Ha, Su Y, Huang D, Liu S, et al. (2007) Spatial variation in organic carbon, nutrients and microbial biomass contents of paddy soils in a hilly red soil region. Frontiers of Agriculture in China 1: 424-429.

118. Corstanje R, Reddy KR, Prenger JP, Newman S, Ogram AV (2007) Soil microbial eco-physiological response to nutrient enrichment in a sub-tropical wetland. Ecological Indicators 7: 277–289.

119. Reddy KR, Wang Y, DeBusk WF, Fisher MM, Newman S (1998) Forms of soil phosphorus in selected hydrologic units of the Florida Everglades. Soil Science Society of America Journal 62: 1134–1147.

120. Oberson A, Friesen DK, Rao IM, Bühler S, Frossard E (2001) Phosphorus transformations in an oxisol under contrasting land-use systems: the role of the soil microbial biomass. Plant and Soil 237: 197–210.

121. Roy S, Singh JS (1994) Consequences of habitat heterogeneity for availability of nutrients in a dry tropical forest. Journal of Ecology 82: 503-509.

122. Singh RS, Srivastava SC, Raghubanshi AS, Singh JS, Singh SP (1991) Microbial C, N and P in dry tropical savanna: Effects of burning and grazing. Journal of Applied Ecology 28: 869-878.

123. Khan KS, Joergensen RG (2006) Microbial C, N, and P relationships in moisture-stressed soils of Potohar, Pakistan. Journal of Plant Nutrition and Soil Science 169: 494-500.

124. Srivastava SC, Lal JP (1994) Effects of crop growth and soil treatments on microbial C, N, and P in dry tropical arable land. Biology and Fertility of Soils 17: 108–114.

125. Agbenin JO, Adeniyi T (2005) The microbial biomass properties of a savanna soil under improved grass and legume pastures in northern Nigeria. Agriculture, Ecosystems & Environment 109: 245–254.

126. Kwabiah AB, Palm CA, Stoskopf NC, Voroney RP (2003) Response of soil microbial biomass dynamics to quality of plant materials with emphasis on P availability. Soil Biology and Biochemistry 35: 207–216.

127. Lajtha K, Schlesinger WH (1988) The biogeochemistry of phosphorus cycling and phosphorus availability along a desert soil chronosequence. Ecology 69: 24–39.

128. Sarig S, Fliessbach A, Steinberger Y (1996) Microbial biomass reflects a nitrogen and phosphorous economy of halophytes grown in salty desert soil. Biology and Fertility of Soils 21: 128–130.

129. Christ MJ, David MB, McHale PJ, Mitchell MJ, Rustad LE, et al. (1997) Microclimatic control of microbial C, N, and P pools in Spodosol Oa horizons. Canadian Journal of Forest Research 27: 1914–1921.

130. Lorenz K, Feger KH, Kandeler E (2001) The response of soil microbial biomass and activity of a Norway spruce forest to liming and drought. Journal of Plant Nutrition and Soil Science 164: 9–19.

131. Joergensen RG, Kübler H, Meyer B, Wolters V (1995) Microbial biomass phosphorus in soils of beech (*Fagus sylvatica* L.) forests. Biology and Fertility of Soils 19: 215–219.

132. Wright CJ, Coleman DC (2000) Cross-site comparison of soil microbial biomass, soil nutrient status, and nematode trophic groups. Pedobiologia 44: 2-23.

133. Meyer K, Joergensen RG, Meyer B (1997) The effects of reduced tillage on microbial biomass C and P in sandy loess soils. Applied Soil Ecology 5: 71–79.

134. Quintern M, Lein M, Joergensen RG (2006) Changes in soil-biological quality indices after long-term addition of shredded shrubs and biogenic waste compost. Journal of Plant Nutrition and Soil Science 169: 488–493.

135. McIntosh PD, Gibson RS, Saggar S, Yeates GW, McGimpsey P (1999) Effect of contrasting farm management on vegetation and biochemical, chemical, and biological condition of moist steepland soils of the South Island high country, New Zealand. Australian Journal of Soil Research 37: 847–866.

136. Ross DJ, Speir TW, Tate KR, Feltham CW (1997) Burning in a New Zealand snow-tussock grassland: effects on soil microbial biomass and nitrogen and phosphorus availability. New Zealand Journal of Ecology 21: 63–71.

137. Saggar S, McIntosh PD, Hedley CB, Knicker H (1999) Changes in soil microbial biomass, metabolic quotient, and organic matter turnover under Hieracium (H. pilosella L.). Biology and Fertility of Soils 30: 232–238.

138. Saggar S, Hedley CB, Giddens KM, Salt GJ (2000) Influence of soil phosphorus status and nitrogen addition on carbon mineralization from 14C-labelled glucose in pasture soils. Biology and Fertility of Soils 32: 209–216.

139. Sarathchandra SU, Perrott KW, Littler RA (1989) Soil microbial biomass: Influence of simulated temperature changes on size, activity and nutrient-content. Soil Biology and Biochemistry 21: 987–993.

140. Turner BL, Bristow AW, Haygarth PM (2001) Rapid estimation of microbial biomass in grassland soils by ultra-violet absorbance. Soil Biology and Biochemistry 33: 913-919.

141. West AW, Ross DJ, Cowling JC (1986) Changes in microbial C, N, P and ATP contents, numbers and respiration on storage of soil. Soil Biology and Biochemistry 18: 141-148.

142. Holland KJ (2006) Fate of nitrogen in alpine tundra [Ph.D. Dissertation]: University of Colorado at Boulder.

143. Kopáček J, Kaňa J, Šantrŭčková H, Picek T, Stuchlık E (2004) Chemical and biochemical characteristics of alpine soils in the Tatra Mountains and their correlation with lake water quality. Water, Air, & Soil Pollution 153: 307–328.

144. Khan KS, Chander K, Hartmann G, Lamersdorf N, Joergensen RG (2007) Sources of heavy metals and their long-term effects on microbial C, N and P relationships in soil. Water, Air, and Soil Pollution 181: 225-234.

145. Saggar S, Parfitt RL, Salt G, Skinner MF (1998) Carbon and phosphorus transformations during decomposition of pine forest floor with different phosphorus status. Biology and Fertility of Soils 27: 197–204.

146. Santrucková H, Vrba J, Picek T, Kopácek J (2004) Soil biochemical activity and phosphorus transformations and losses from acidified forest soils. Soil Biology and Biochemistry 36: 1569–1576.

147. Chu H, Grogan P (2009) Soil microbial biomass, nutrient availability and nitrogen mineralization potential among vegetation-types in a low arctic tundra landscape. Plant and Soil 329: 411-420.

148. Lagerström A, Esberg C, Wardle DA, Giesler R (2009) Soil phosphorus and microbial response to a long-term wildfire chronosequence in northern Sweden. Biogeochemistry 95: 199-213.

149. Larsen KS, Grogan P, Jonasson S, Michelsen A (2007) Respiration and microbial dynamics in two subarctic ecosystems during winter and spring thaw: effects of increased snow depth. Arctic, Antarctic, and Alpine Research 39: 268–276.
